# BET inhibitors reduce tumor growth in preclinical models of gastrointestinal gene signature–positive castration-resistant prostate cancer

**DOI:** 10.1172/JCI180378

**Published:** 2025-06-24

**Authors:** Shipra Shukla, Dan Li, Woo Hyun Cho, Dana M. Schoeps, Holly M. Nguyen, Jennifer L. Conner, Marjorie L. Roskes, Anisha Tehim, Gabriella Bayshtok, Mohini R. Pachai, Juan Yan, Nicholas A. Teri, Eric Campeau, Sarah Attwell, Patrick Trojer, Irina Ostrovnaya, Anuradha Gopalan, Ekta Khurana, Eva Corey, Ping Chi, Yu Chen

**Affiliations:** 1 Human Oncology and Pathogenesis Program, Memorial Sloan Kettering Cancer Center, New York, New York, USA.; 2 Department of Urology, University of Washington, Seattle, Washington, USA.; 3 Weill Cornell Medicine, New York, New York, USA.; 4 Weill Cornell Medical College, New York, New York, USA.; 5 Zenith Epigenetics, Calgary, Alberta, Canada.; 6 Triana Biomedicines, Waltham, Massachusetts.; 7 Department of Epidemiology Biostatistics,; 8 Department of Pathology and Laboratory Medicine, and; 9 Department of Medicine, Memorial Sloan Kettering Cancer Center, New York, New York, USA.

**Keywords:** Cell biology, Genetics, Oncology, Drug therapy, Epigenetics

## Abstract

A subgroup (~20%–30%) of castration-resistant prostate cancer (CRPC) aberrantly expresses a gastrointestinal (GI) transcriptome governed by 2 GI-lineage-restricted transcription factors, HNF1A and HNF4G. In this study, we found that expression of GI transcriptome in CRPC correlated with adverse clinical outcomes to androgen receptor (AR) signaling inhibitor treatment and shorter overall survival. Bromo- and extraterminal domain inhibitors (BETi) downregulated HNF1A, HNF4G, and the GI transcriptome in multiple CRPC models, including cell lines, patient-derived organoids, and patient-derived xenografts, whereas AR and the androgen-dependent transcriptome were largely spared. Accordingly, BETi selectively inhibited growth of GI transcriptome-positive preclinical models of prostate cancer. Mechanistically, BETi inhibited BRD4 binding at enhancers globally, including both AR and HNF4G bound enhancers, while gene expression was selectively perturbed. Restoration of HNF4G expression in the presence of BETi rescued target gene expression without rescuing BRD4 binding. This suggests that inhibition of master transcription factors expression underlies the selective transcriptional effects of BETi.

## Abstract 2

BET inhibitors show promising efficacy in a subset of castration-resistant prostate cancer marked by overexpression of a gastrointestinal gene signature in multiple preclinical models.

## Introduction

Lineage plasticity is increasingly being appreciated as a mechanism to evade targeted therapy by cancer cells of multiple origins and lineages. Examples include prostate cancer and EGFR-mutant lung cancer, in which adenocarcinomas transdifferentiate into neuroendocrine cancers under select pressure of targeted therapy. In this process, cancer cells lose dependence on the initial tumor drivers, androgen receptor (AR) in prostate cancer, and EGFR and other oncogenic RTKs in lung cancer (1–3). However, in contrast to a complete switch to neuroendocrine lineage, a significant fraction of prostate adenocarcinoma also exists in a heterogeneous and plastic state where cancer cells acquire features of alternate cellular lineages and states such as stem cells, basal cells, and mesenchymal cells (4–7). This poses a challenge in targeted therapy because (a) multiple dependencies exist in such tumors and (b) therapeutic targeting of the primary lineage may augment the process toward a complete lineage switch (8). Therefore, combination therapies targeting more than 1 lineage/pathway may be more successful in such cases.

We have previously reported the activation of a gastrointestinal (GI) lineage transcriptome governed by aberrant expression of master regulators HNF1A and HNF4G in a significant fraction of castration-resistant prostate cancer (CRPC) (9). HNF4G and HNF1A form a regulatory circuit where they influence each other’s expression. Exogenous expression of either HNF4G or HNF1A is sufficient to express the GI transcriptome in LNCaP cells that do not express either transcription factor. Expression of this aberrant GI transcriptome mediates resistance to enzalutamide (9). In the present study, using 2 different metastatic CRPC (mCRPC) data sets, we show that increased GI transcriptome expression in patient tumors is associated with a shorter time on treatment with androgen receptor signaling inhibitors (ARSI) as well as a shorter overall survival. We hypothesized that inhibition of this transcriptome would provide therapeutic benefits in patients. Our studies revealed that inhibitors against bromodomain and extraterminal (BET) family member proteins efficiently inhibit GI transcriptome expression by directly targeting *HNF1A* and *HNF4G* transcription. Finally, we show the selective growth inhibitory effect elicited by BET inhibitors (BETis) either alone or in combination with enzalutamide on GI-transcriptome-expressing preclinical CRPC models, including patient-derived organoids and xenografts.

## Results

### Aberrant expression of GI transcriptome in CRPC correlates with adverse clinical outcomes to ARSI treatment.

The expression of GI transcriptome is governed by master regulators HNF1A and HNF4G and it is more prevalent in mCRPC compared with localized prostate cancer across multiple gene-expression data sets (9). Experimentally, exogenous expression of HNF4G in prostate cancer cells leads to expression of the GI transcriptome and resistance to AR pathway inhibition. These data suggest a causal relationship between the expression of GI transcriptome and resistance to AR-targeted therapy (9).

Here, we sought to quantify the level of GI transcriptome expression and correlate it with clinical outcomes. We derived an HNF signature comprising HNF1A, HNF4G, and their 9 strong direct downstream targets and an HNF score derived from the summed *z* scores of their gene expression. Correlation analysis performed on 2 clinical gene expression data sets showed that the HNF score was significantly correlated with HNF1A and HNF4G expression (Supplemental Figure 1, A and B; supplemental material available online with this article; https://doi.org/10.1172/JCI180378DS1). The HNF score also strongly correlated with the broader prostate cancer-gastrointestinal (PCa_GI) signature sum *z* score (Supplemental Figure 1, A and B). The PCa_GI signature was previously defined and derived from correlation with SPINK1 in primary prostate cancer (9). We applied the HNF score to analyze 2 RNA-Seq data sets of CRPC tumors from patients treated with ARSIs.

**Figure 1:**
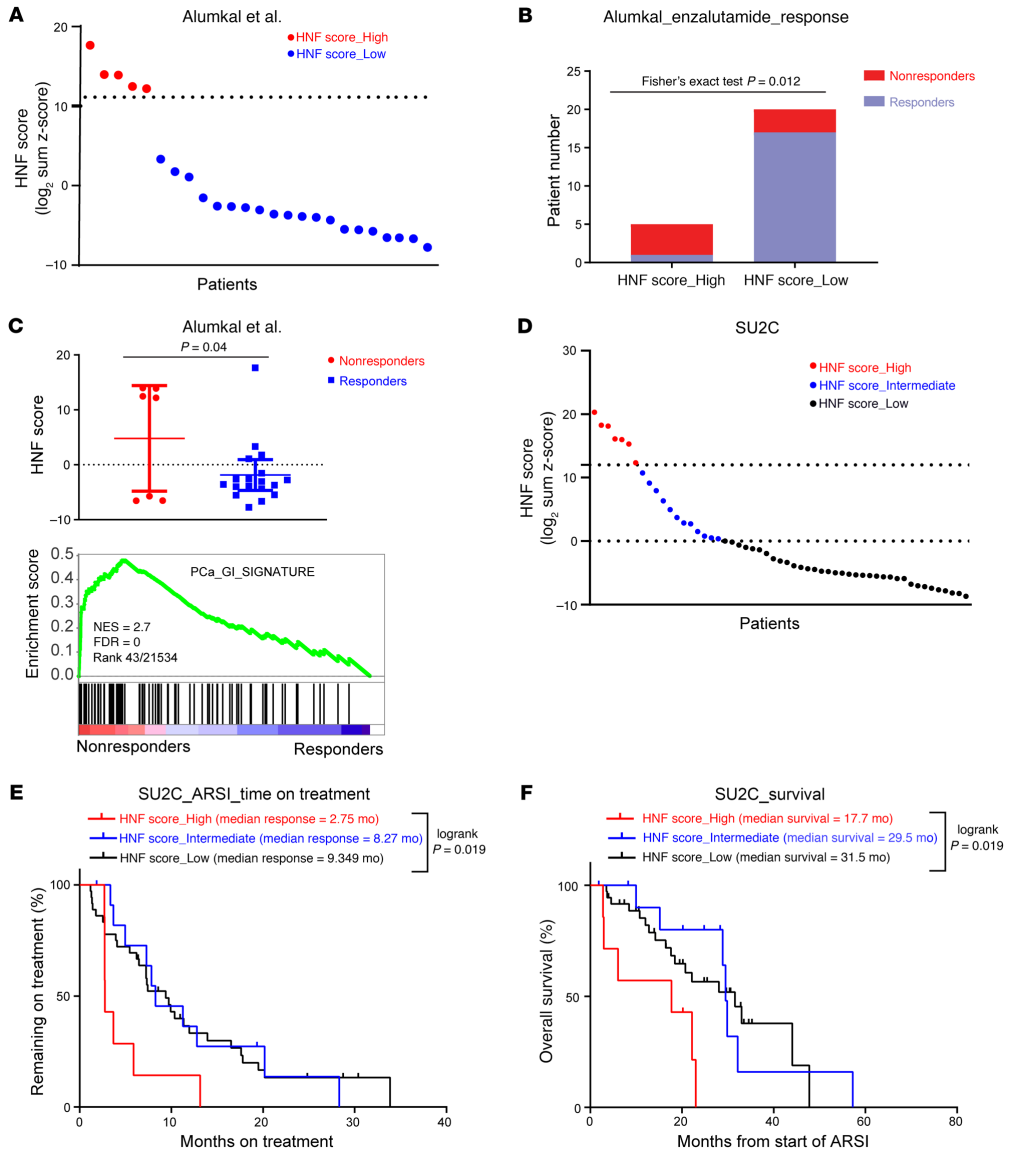
A high HNF score in CRPC correlates with adverse clinical outcomes. (**A**) Patient stratification based on HNF scores in the Alumkal et al. data set (10). Each dot represents 1 patient. The HNF score was calculated as the log2 sum *z* score of mRNA expression of 11 genes. A sum *z* score ≥ 12 was annotated as a high HNF score and < 12 as a low HNF score. (**B**) Enzalutamide response of patient tumors with high and low HNF scores. Statistical significance was determined using Fisher’s exact test. (**C**) Comparison of HNF scores between enzalutamide nonresponders and responders (top) and GSEA plot of PCa_GI gene signature (bottom) in enzalutamide nonresponders compared with responders. *P* values determined by unpaired, 2-tailed *t* test. NES, normalized enrichment score. (**D**) Patient stratification based on HNF score expression in the SU2C data set. Each dot represents 1 patient. Tumors with a sum *z* score of ≥ 12 were annotated as expressing a high HNF score; a sum *z* score of ≤ 0 was a low HNF score; and a value between 0 and 12 was intermediate HNF_score. (**E**) Kaplan-Meier curve comparing ARSI outcome measures among the 3 groups stratified by HNF scores. *P* values were determined by log-rank (Mantel-Cox) test. (**F**) Kaplan-Meier curve comparing overall survival outcomes among the 3 groups stratified by HNF scores. *P* values were determined by log-rank (Mantel-Cox) test.

The clinical trial Genetic and Molecular Mechanisms in Assessing Response in Patients with Prostate Cancer Receiving Enzalutamide Therapy (ClinicalTrials.gov NCT02099864) prospectively enrolled 36 taxane- and abiraterone-naive patients with mCRPC to receive treatment with enzalutamide (10). Response was defined as a 50% decline in prostate-specific antigen (PSA) level after 12 weeks of treatment. Among the 25 patients with pretreatment RNA-Seq data, we found that 20% of tumors (*n* = 5) had a higher HNF score (*z* > 12) than the rest. We used this cutoff to define HNF score_High tumors (Figure 1A). Notably, 4 of 5 patients with HNF score_High tumors, but only 3 of 20 patients with HNF score_Low tumors, did not respond to enzalutamide treatment (Fisher’s exact test *P* = 0.012) (Figure 1B). Alternatively, 4 of 7 nonresponders had a high HNF score compared with 1 of 18 of responders (Figure 1C and Supplemental Figure 1C). Global transcriptome analysis showed a significant upregulation of many GI lineage genes, such as HNF1A, MUC13, UGT2B4, MIA2, and NR1H4, in enzalutamide nonresponders compared with responders (Supplemental Figure 1D).

To identify pathways enriched in nonresponding tumors, we performed gene set enrichment analysis (GSEA) comparing nonresponders and responders using the Molecular Signatures Database (MSigDB) comprising > 20,000 gene sets and our custom gene sets. We found our previously defined PCa_GI_signature gene set as well as a gene set comprising HNF1A targets were significantly enriched in enzalutamide nonresponders (Figure 1C, Supplemental Figure 1E, and Supplemental Table 1A). The other top significantly enriched gene sets in nonresponders were related to metastasis and immune functions (Supplemental Figure 1F and Supplemental Table 1A).

To understand the correlation between HNF and AR signatures, we derived an AR score as the sum *z* score of AR target genes combined from 2 different AR signatures (11, 12). We noticed that the 5 tumors with high HNF scores had lower AR scores, although this correlation did not reach statistical significance (Supplemental Figure 1G). Next, we asked if any CRPC subtypes are specifically enriched for high HNF score–expressing tumors. Using our previously published methodology, our analysis revealed that high HNF-score tumors showed characteristics of the AR subtype (Supplemental Figure 1H and Supplemental Table 2) (7).

We next analyzed the RNA-Seq data of patients with mCRPC from SU2C International Dream Team and calculated the HNF score for patients for whom the overall survival and time on ARSI treatment information was available (13, 14). We analyzed ARSI-naive patients going onto ARSI therapy (*n* = 50). We ranked patients based on the tumor HNF scores, annotating them into 3 categories: patients with a sum *z* score value of > 12 as in the Alumkal et al. data set (10) were categorized as HNF score_High, whereas patients with a sum *z* score of 0 or less were categorized as HNF score_Low. The remaining patients were categorized as HNF score_Intermediate (Figure 1D). Kaplan-Meier analysis revealed that the patients categorized as HNF score_High had the shortest median time on ARSI (Figure 1E). To investigate whether the poor response to ARSI would translate to shorter overall survival of these patients, we performed a Kaplan-Meier survival analysis and found that HNF score_High patients had a significantly shorter overall survival as compared with the other 2 cohorts (Figure 1F). These data suggest that increased expression of the GI transcriptome in patients is associated with worse clinical outcomes in patients with CRPC. In this data set, the HNF score in tumors correlated negatively with the AR score (Supplemental Figure 1I). Analysis of CRPC subtype classification revealed the stem cell–like SCL subtype to be enriched in HNF score_High tumors, whereas the AR-dependent subtype were predominant in HNF score_Low tumors (Supplemental Figure 1J and Supplemental Table 3).

### BET inhibition downregulates GI transcriptome in CRPC.

**Figure 2:**
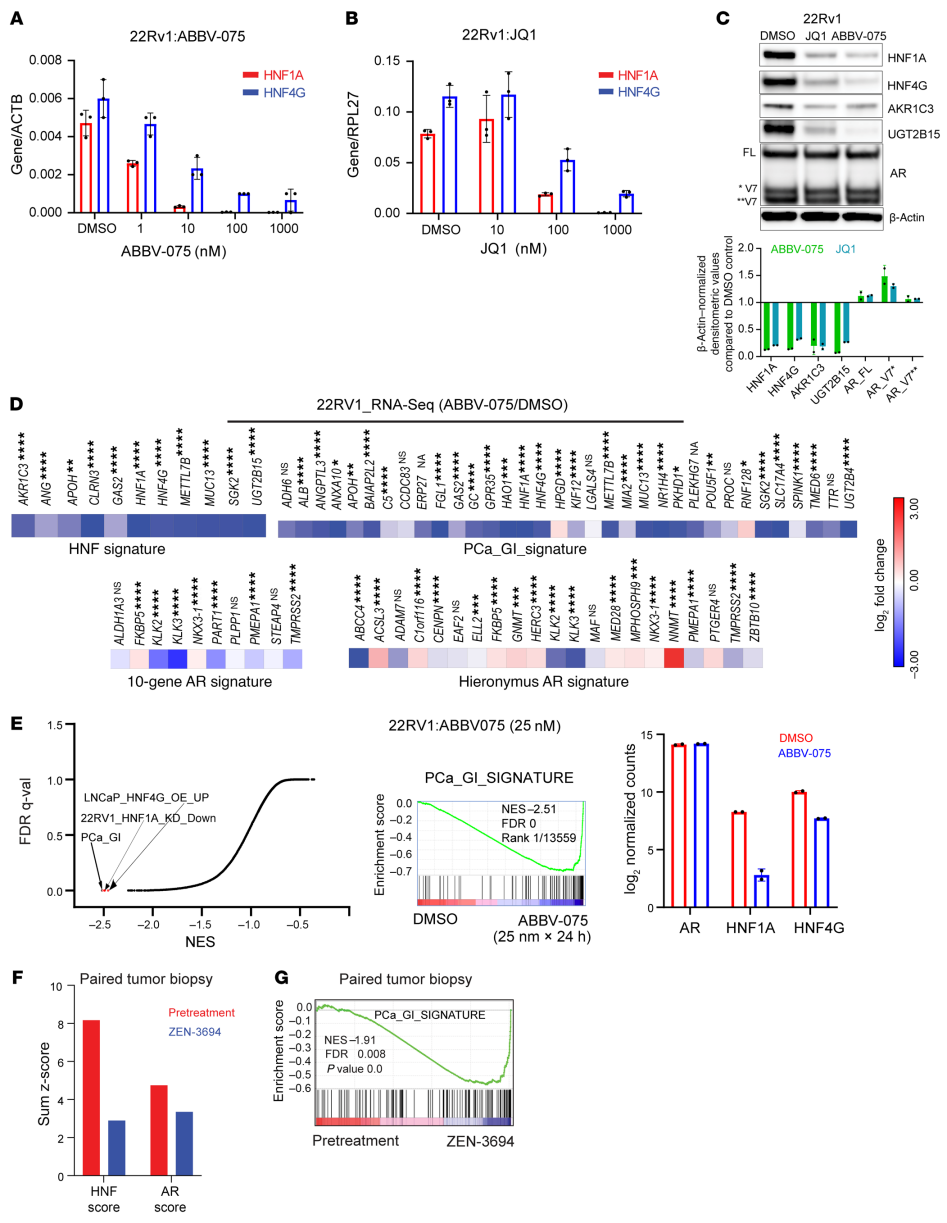
BETis downregulate the expression of *HNF4G* and *HNF1A* and their transcriptional signature. (**A**) qRT-PCR showing expression of HNF1A after 4 hours of treatment with ABBV-075 and JQ1 at indicated doses. (**B**) qRT-PCR showing expression of HNF4G after 4 hours of treatment with ABBV-075 and JQ1 at indicated doses. (**C**) A representative immunoblot of 22Rv1 cells treated with JQ (0.5 μM), ABBV-075 (50 nM), and DMSO control for 24 hours against the indicated proteins (top). Bar graph (bottom) showing fold change in β-actin normalized band intensities of JQ1- and ABBV-075–treated samples over DMSO controls (*n* = 2). (**D**) Heat map of RNA-Seq expression of HNF signature genes in 22Rv1 cells after treatment with 25 nM ABBV-075 for 24 hours (top). (Bottom) The 2 heat maps show the modulation of AR target genes with ABBV-075 treatment using 2 different AR gene signatures. Data are plotted as the log_2_ difference in gene expression between ABBV-075– and DMSO-treated cells. Unadjusted *P* values are shown: *P < 0.05, **P < 0.01, ***P < 0.001, and ****P < 0.0001. (**E**) Global representation of GSEA analysis of RNA-Seq gene expression data set of 22RV1 cells treated with 25 nM ABBV-075 for 24 hours. The *x*-axis shows the normalized enrichment score, and *y*-axis is the FDR *q*-value (q-val). The PCa_GI and the HNF1A- and HNF4G-regulated gene sets are indicated in red. A GSEA plot of PCa_GI gene signature is shown (middle). (Right) A bar diagram shows the expression of AR, HNF1A, and HNF4G. NES, normalized enrichment score. (**F**) Modulation of HNF and AR scores by BETi ZEN-3694 in paired tumor biopsy specimens of patient 101047. (**G**) GSEA plots of PCa_GI gene signature in ZEN-3694–treated tumors compared with pretreated tumor. NES, normalized enrichment score.

Previously, we showed that HNF4G is required for maintaining open chromatin regions and active transcription-associated epigenetic modifications such as H3K4me1 and H3K27ac at its target genes. Members of the BET family proteins, BRD2, BRD3, and BRD4 are epigenetic readers. They bind to acetylated histones through their bromodomains and facilitate the assembly of active transcriptional complexes. In the absence of selective inhibitors against HNF1A and HNF4G, we explored the impact of targeting BET proteins on GI transcriptome expression. We used 2 BETis, ABBV-075 (mivebresib) and JQ1, in experiments performed on 22Rv1 cells that express the GI transcriptome. Treatment with either inhibitor for 4 hours led to a dose-dependent decrease in transcripts of *HNF1A* and *HNF4G* (Figure 2 A and B), whereas the *AR* transcript was only modestly inhibited at high concentrations (Supplemental Figure 2A). Immunoblot analysis at 24 hours after treatment showed reduced protein levels of HNF1A and HNF4G, as well as their downstream targets AKR1C3 and UGT2B15, whereas AR protein levels remained unchanged (Figure 2C and Supplemental Figure 2B). These cells also showed a dose-dependent decrease in cell viability when treated with ABBV-075 and JQ1 (Supplemental Figure 2C).

To analyze the effect of BET inhibition on global gene expression, we performed RNA-Seq of 22Rv1 cells treated with 25 nM ABBV-075 for 24 hours. The ABBV-075 treatment led to a downregulation of the HNF signature as well as the broader PCa_GI signature (Figure 2D). However, the effect of ABBV-075 on the AR transcriptome varied, as assessed using 2 different AR signatures with some genes, such as *KLK3*, that were strongly downregulated, whereas *FKBP5* and *NKX3-1* were upregulated (11, 12) (Figure 2D). We next performed GSEA on RNA-Seq data obtained from DMSO and ABBV-075 treated cells. The topmost downregulated gene sets in ABBV-075–treated cells included the PCa_GI signature and gene sets regulated by HNF4G and HNF1A (Figure 2E, Supplemental Table 1B).

We further analyzed 3 publicly available RNA-Seq data sets on BETi (JQ1 and ABBV075) treatment in 22Rv1 cells (15–17). GSEA of the RNA-Seq data showed that for both JQ1 and ABBV-075, gene sets regulated by HNF1A and HNF4G, and the PCa_GI signature, were among the most significantly downregulated gene sets. Consistent with our observations, BETi treatment caused downregulation of *HNF1A* and *HNF4G* transcriptions but not *AR* transcript (Supplemental Figure 2, D–F, and Supplemental Table 1, C–E).

BETis, including ZEN-3694 and NUV-868, are being evaluated in clinical trials in various cancer types, including prostate cancer (ClinicalTrials.gov NCT02705469, NCT04471974, NCT04986423, NCT02711956, and NCT05252390). We took advantage of a recently completed phase 1b/2a clinical trial of the BETi ZEN-3694 on a cohort of patients with mCRPC (18). Gene expression data from before and after ZEN-3694 treatment were available for 4 patients. Among them, a pretreatment biopsy specimen from patient 101047 exhibited a high HNF score. RNA-Seq analysis performed on the paired biopsy tumor samples of patient 101047 revealed downregulation of HNF score after ZEN-3694 treatment compared with the pretreatment biopsy score, although the AR score was only modestly downregulated upon BET inhibition (Figure 2F). GSEA showed the downregulation of the PCa_GI_signature and HNF4G target gene sets by ZEN-3694 in the post-treatment biopsy specimen (Figure 2G and Supplemental Figure 2G). ZEN-3694 treatment caused downregulation of HNF1A and HNF4G, but not AR, in the post-treatment biopsy specimen (Supplemental Figure 2H and Supplemental Table 1F). These anecdotal data are consistent with our data from cell lines, patient-derived organoids and patient-derived xenografts (PDX) that BET inhibition inhibits transcription of HNF1A, HNF4G, and the GI signature.

### Inhibition of HNF4G transcription principally accounts for BETi-mediated inhibition of GI transcriptome.

**Figure 3:**
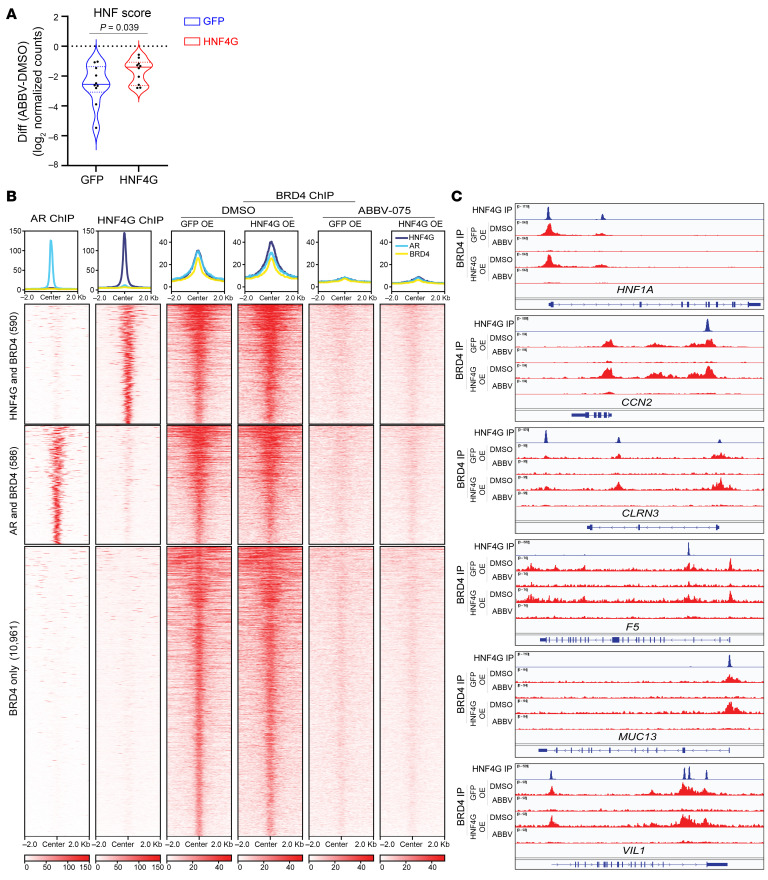
Inhibition of HNF4G transcription accounts for BETi-mediated inhibition of GI transcriptome. (**A**) Violin plot of log_2_ fold changes in expression of HNF score genes by ABBV-075 treatment in 22Rv1 cells exogenously expressing GFP or HNF4G compared with DMSO control. The median is represented by a solid line, and the first and third quartiles are indicated by dashed lines with all dots plotted. Statistical analysis was performed using a 2-tailed paired *t* test. “Diff” represents the difference in log_2_-normalized gene expression counts of HNF score genes between ABBV-075–treated and DMSO-treated samples in GFP- and HNF4G-overexpressing cells. (**B**) Histograms (top) show the average normalized tag counts of AR and HNF4G in parental 22Rv1 cells and that of BRD4 in GFP- or HNF4G-expressing 22Rv1 cells treated with ABBV-075 or DMSO at top 1,000 HNF4G, 1,000 AR binding sites, and BRD4-only enhancer binding sites. Heat map shows the tag densities of HNF4G, AR, and that of BRD4 at HNF4G (top) or AR (middle) binding sites. Bottom panel show the tag densities of BRD4 at 10,961 BRD4-only sites in GFP- or HNF4G-expressing 22Rv1 cells treated with ABBV-075 or DMSO. (**C**) ChIP-Seq profiles of HNF4G in parental 22Rv1 cells and BRD4 (DMSO treatment), and BRD4 (ABBV-075 treatment) in GFP- or HNF4G-expressing 22Rv1 cells at selected HNF4G target genes loci: *HNF1A*, *CCN2*, *CLRN3*, *F5*, *MUC13*, and *VIL1* in top to bottom order.

We asked whether the preferential inhibition of GI transcriptome over AR-regulated transcriptome by BETi treatment is due to downregulation of master transcription factors HNF4G and HNF1A but not of AR. To explore this possibility, we generated 22Rv1 derivatives that exogenously express HNF4G (HNF4G OE) or GFP (GFP OE) from the murine stem cell virus promoter that is not repressed with ABBV-075 (Supplemental Figure 3, A and B). We then treated GFP OE and HNF4G OE cells with ABBV-075 (25 nM) or DMSO for 24 hours and performed RNA-Seq analysis. We compared the effect of ABBV-075 treatment on the expression of HNF score genes between GFP or HNF4G expressing cells, using DMSO treatment as a control. We observed that restoring the expression of HNF4G can largely reverse the ABBV-075–mediated downregulation of HNF-score signature genes as well as of the other HNF4G targets (Figure 3A and Supplemental Figure 3C). Examination of individual genes shows partial (*HNF1A*) to almost complete (*CCN2*, *CLRN3*, *VIL1*) rescue of transcriptional inhibition (Supplemental Figure 3C). We performed HNF4G ChIP-Seq in both GFP- and HNF4G-overexpressing cells treated with ABBV-075 or DMSO control. ABBV-075 treatment in GFP-expressing cells led to a global decrease in HNF4G binding (Supplemental Figure 3D). In the HNF4G OE cells, HNF4G binding was maintained globally with ABBV-075 treatment consistent with restoration of HNF4G protein levels in these cells (Supplemental Figure 3, B and D).

Because BRD4 is the most extensively characterized member of BET family proteins, we next examined the requirement of BRD4 at the loci of these transcriptionally rescued genes. We performed BRD4 ChIP-Seq in GFP OE and HNF4G OE cells treated with ABBV-075 (25 nM for 4 hours) or DMSO. We examined BRD4 binding at BRD4 peaks that overlapped with previously defined top 1,000 HNF4G peaks (*n* = 590), top 1,000 AR peaks (*n* = 586), and nonoverlapping BRD4 peaks (*n* = 10,961). Exogenous expression of HNF4G led to a modest increase of BRD4 binding at HNF4G binding sites but not at AR binding sites or nonoverlapping sites. ABBV-075 treatment broadly displaced BRD4 from chromatin at all BRD4 peaks, and exogenous HNF4G expression did not rescue BRD4 binding (Figure 3B). Examination of ChIP-Seq tracks of selected genes shown in Supplemental Figure 3C reveals a similar level of BRD4 displacement with ABBV-075 treatment in between GFP OE and HNF4G OE cells despite their continued transcription in HNF4G OE cells (Figure 3C). These data suggest BRD4 is an accessory factor rather than the primary factor in controlling gene expression regulation. Restoration of HNF4G binding mitigates the transcriptional effects of BRD4 displacement at its target genes.

To compare the effects of HNF1A and HNF4G overexpression on BETi-mediated inhibition of target gene transcription, we overexpressed HNF1A, HNF4G, and RFP in 22Rv1 cells and performed qRT-PCR on select target genes after treatment with 25 nM ABBV-075 and 250 nM JQ1 for 24 hours. As expected, exogenous expression of HNF1A and HNF4G under the murine stem cell virus promoter led to overexpression of the respective transcripts that were insensitive to BETi. HNF4G OE led to upregulation of all transcripts tested (*HNF1A*, *UGT2B15*, *SGK2*, *AKR1C3*, *APOH*, *ANG*, *CLRN3*, *MUC13*, and *METTL7B*). Some transcripts maintained some BETi sensitivity (e.g., *HNF1A*, *MUC13*) and some were completely rescued (e.g., *AKR1C3*, *UGT2B15*). HNF1A OE upregulated *HNF4G* and most downstream gene expression. We observed almost complete transcriptional rescue of genes such as *UGT2B15, AKR1C3*, and *APOH*; a partial rescue of *HNF4G*, *SGK2*, and *CLRN3* expression, and no rescue of *MUC13* and *METTL7B* expression with BETi treatments. In contrast to HNF1A, HNF4G OE showed a stronger rescue of *CLRN3*, *MUC13*, and *METTL7B* transcription but a weaker rescue of *APOH* transcription, suggesting target gene selectivity (Supplemental Figure 3E).

We next performed similar studies in MSK-PCa10, an HNF-high organoid model in which we overexpressed HNF4G, HNF1A, and RFP and treated with ABBV-075 (25 nM) or JQ1(250 nM) or DMSO control for 24 hours. We observed a similar and selective transcriptional rescue of HNF-regulated genes by HNF1A (*UGT2B15*, *SGK2*) and HNF4G (*CLRN3*) (Supplemental Figure 3F). These data broadly suggest the downregulation of master transcription factors underlies the selectivity of BETi transcriptional inhibition despite the broad displacement of BET proteins from chromatin (9, 15).

### GI transcriptome-positive prostate cancer models exhibit increased sensitivity to BETis.

**Figure 4:**
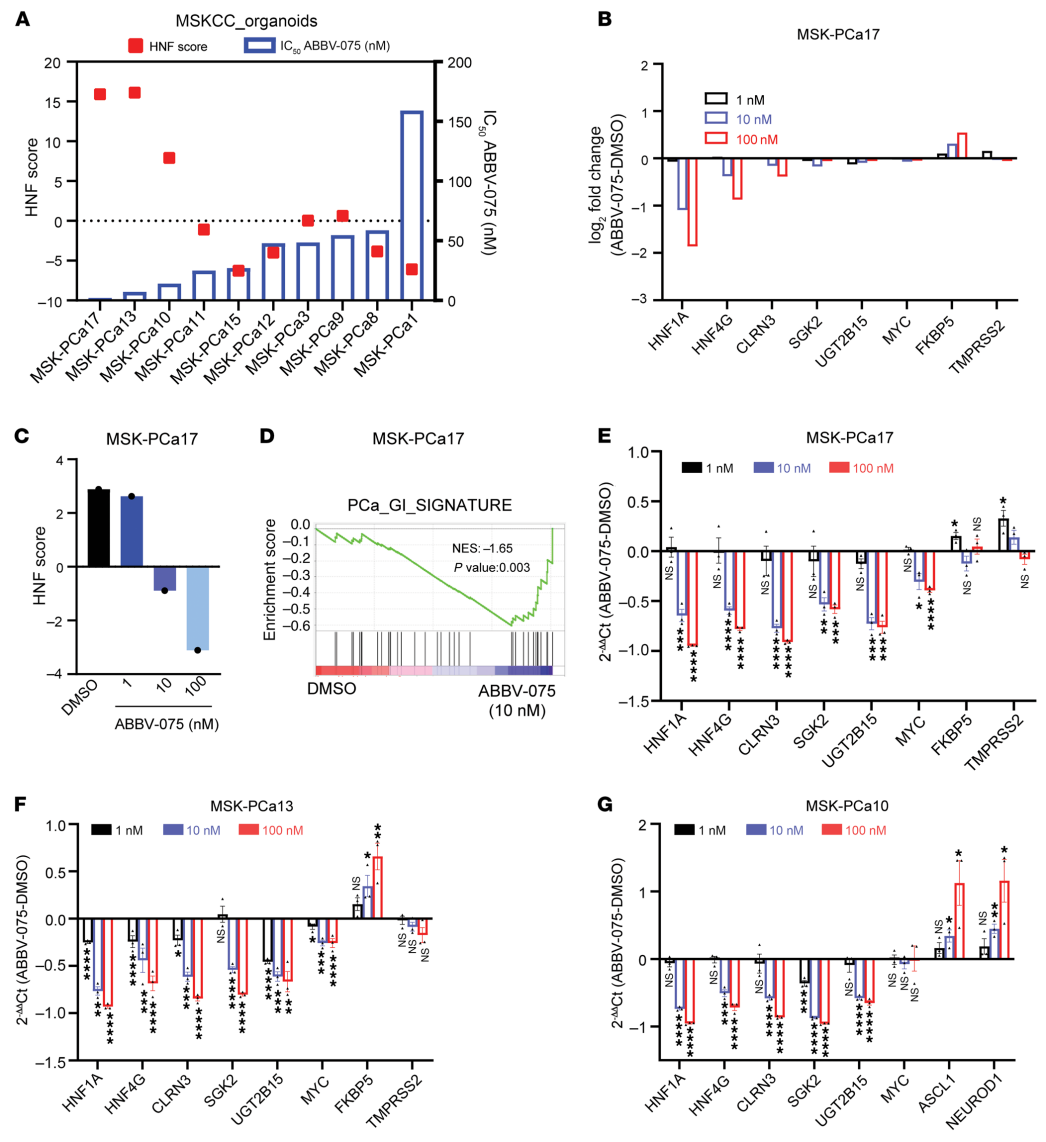
Patient-derived organoids with high HNF scores show increased sensitivity to BETi-mediated growth inhibition. (**A**) IC_50_ of ABBV-075 in a panel of patient-derived tumor biopsy specimens grown as organoids. The left-side *y*-axis plots the HNF scores of each organoid and the right-side *y*-axis shows the IC_50_ values. (**B**) RNA-Seq gene expression changes of selected genes at different doses of ABBV-075 treatment of MSK-PCa17 cells compared with DMSO control. Data are presented as the log2-fold difference in expression (ABBV-075 vs DMSO). (**C**) A bar graph showing changes in HNF score expression in MSK-PCa17 cells at different dosesh of ABBV-075 treatment compared with DMSO control. (**D**) GSEA analysis indicating the negative enrichment of PCa_GI gene signature gene set in MSK-PCa17 cells treated with ABBV-075 (10 nM) compared with DMSO control. NES, normalized enrichment score. (**E**) qRT-PCR showing expression of selected genes after 4 hours of treatment with ABBV-075 at indicated doses in MSK-PCa17 cells (*n* = 3). (**F**) qRT-PCR showing expression of selected genes after 4 hours of treatment with ABBV-075 at indicated doses in MSK-PCa13 cells (*n* = 3). (**G**) qRT-PCR showing expression of selected genes after 4 hours of treatment with ABBV-075 at indicated doses in MSK-PCa10 cells (*n* = 3). *P* values were obtained from an unpaired, 2-tailed *t* test. *P < 0.05, **P < 0.01, ***P < 0.001, and ****P < 0.0001.

To examine the effect of BET inhibition on the growth of GI transcriptome-positive prostate cancer, we treated with ABBV-075 10 prostate cancer organoids derived from patients with mCRPC (7, 19). Notably, we observed that organoids with a high HNF score—MSK-PCa17, MSK-PCa13, and MSK-PCa10—had the lowest IC_50_ to ABBV-075 treatment, suggesting high sensitivity (Figure 4A and Supplemental Figure 4A). MSK-PCa17 cells had the lowest IC_50_ to ABBV-075 (IC_50_ < 2 nM) among all the organoids.

To identify important genes/pathways perturbed by ABBV-075, we performed RNA-Seq on MSK-PCa17 cells treated with ABBV-075 at 3 different concentrations (1 nM, 10 nM, and 100 nM) for 4 hours. Our results showed that *HNF1A* and *HNF4G* were downregulated in a dose-dependent manner, along with other signature GI transcriptome genes such as *CLRN3*, *SGK2*, and *UGT2B15* (Figure 4B). ABBV-075 treatment decreased the HNF score and downregulated the broader PCa_GI_signature (Figure 4, C and D). We next performed qRT-PCR analysis in these cells with the same treatment and observed a dose-dependent decrease in expression of HNF1A, HNF4G, and downstream targets (Figure 4E). In MSK-PCa13 cells, the second most sensitive line, qRT-PCR analysis demonstrated a dose-dependent decrease in HNF1A, HNF4G, and downstream target genes transcript levels with ABBV-075 treatment (Figure 4F). MSK-PCa10, a neuroendocrine prostate cancer (NEPC) organoid with a high HNF score, showed sensitivity to BET inhibition. qRT-PCR analysis revealed no decrease in important NEPC lineage genes such as *ASCL1* and *NEUROD*. However, HNF1A and HNF4G and their downstream targets expression were suppressed by ABBV-075 in these cells (Figure 4G). These data suggest organoids with a high HNF score are sensitive to growth inhibition by ABBV-075, emphasizing the potential relevance of the GI transcriptome expression to BET inhibition response. The observed downregulation of key genes and pathways associated with the GI transcriptome supports the potential therapeutic efficacy of BET inhibition in this context. We also performed cell viability studies in organoids using JQ1 and noticed that, like ABBV-075, the most sensitive models to JQ1-mediated growth inhibition expressed high HNF scores (Supplemental Figure 4, B and C).

**Figure 5:**
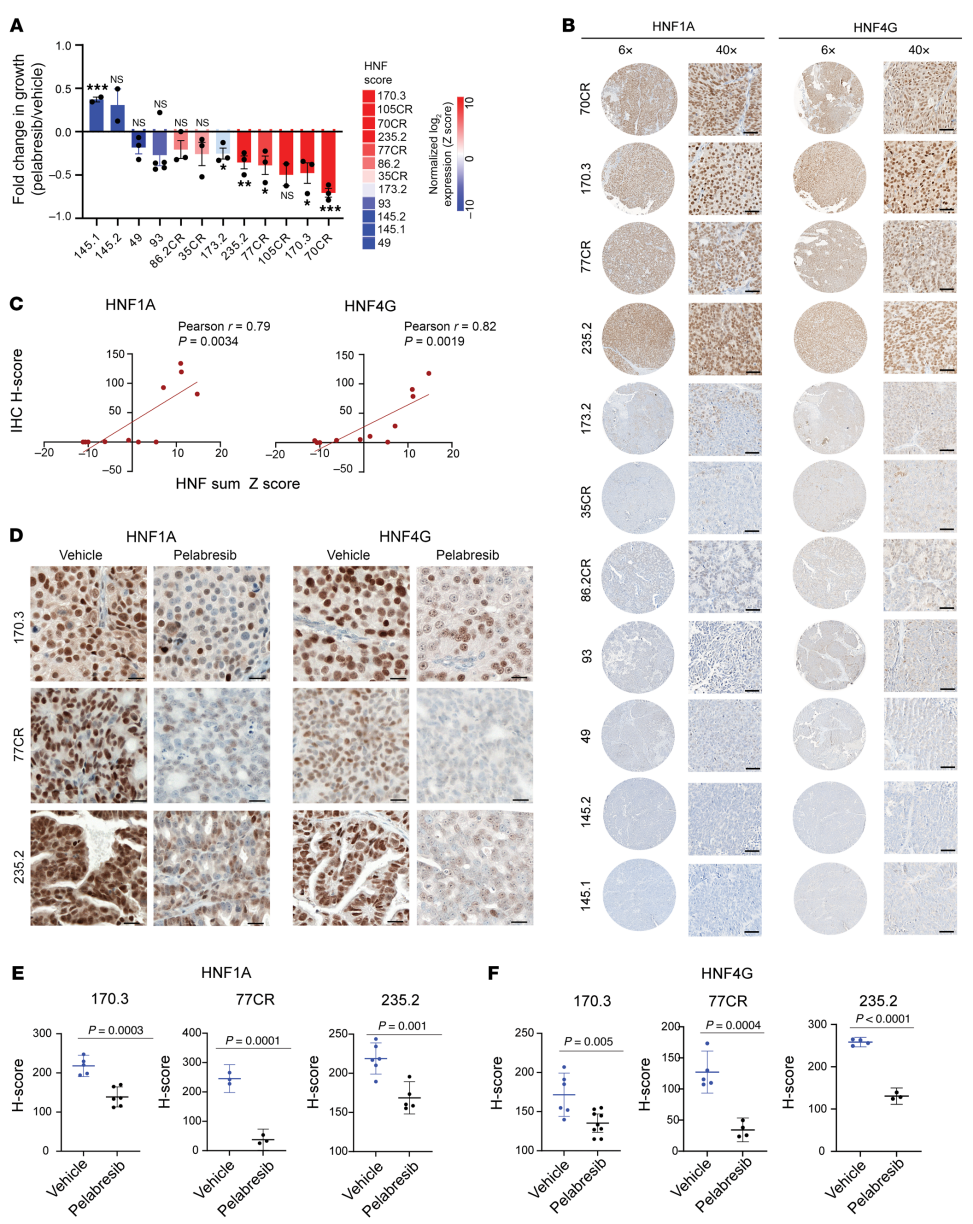
CRPC PDXs expressing high HNF score are sensitive to BET inhibition. (**A**) Treatment response of LuCaP PDXs when treated with pelabresib (30 mg/kg) or vehicle (1% carboxymethyl cellulose) twice a day. Treatment was started when tumors reached a volume of approximately 100 mm^3^. Data are plotted as the fold change in tumor volume between pelabresib and vehicle-treated tumors after 4 weeks of treatment. *n* = 2–5 for different PDX; data are mean ± SEM. *P* values were obtained from a 2-tailed unpaired *t* test. *P < 0.05, **P < 0.01, and ***P < 0.001. The HNF score of PDXs is shown on top of the graph. (**B**) Representative images of HNF4G and HNF1A IHC in LuCaP PDX tissue microarrays at a lower (×6) and higher magnification (×40; scale bar: 50 μm) (*n* = 3). (**C**) Correlation between 11-gene HNF sum *z* score and HNF4G and HNF1A IHC stain-based H scores of each PDX shown in **B**. Pearson’s correlation coefficient and *P* values are indicated on each plot. (**D**) Representative images of HNF4G and HNF1A IHC in selected LuCaP PDXs when treated with pelabresib or vehicle control. Scale bar: 20 μm. (**E**) Scatter plots of HNF1A IHC H-scores in vehicle- and pelabresib-treated PDX tumors. Data are mean ± SEM. *P* values were obtained from a 2-tailed unpaired *t* test. (**F**) Scatter plots of HNF1A IHC H scores in vehicle- and pelabresib-treated PDX tumors. Data are mean ± SEM. *P* values were obtained from a 2-tailed unpaired *t* test.

Next, we did a preliminary screen to assess the response to BETi in vivo using a panel of 12 LuCaP PDXs that are well annotated and represent the varied clinical spectrum of CRPC (20, 21). We chose pelabresib (CPI-0610) for in vivo studies because it has favorable pharmacokinetics and pharmacodynamics properties and is in late-stage clinical development (22). We treated each PDX with pelabresib or vehicle for 4 weeks. Fold changes in tumor volume were determined by comparing the pelabresib-treated group and the vehicle-treated group after the 4-week treatment period. HNF scores for each PDX were calculated using baseline RNA-Seq data. We observed that PDXs with higher HNF scores were more sensitive to the growth-inhibitory effects of pelabresib (Figure 5A). We also performed IHC staining of HNF1A and HNF4G on tissue microarrays of LuCaP PDXs to validate the mRNA-based HNF score annotations. We observed that PDXs with high HNF scores showed strong nuclear staining for both HNF1A and HNF4G (Figure 5B). The HNF1A and HNF4G staining intensities were quantified to obtain an IHC H score for each PDX, and the HNF score and IHC H score showed a strong correlation (Figure 5C). We next examined the effect of pelabresib treatments on HNF1A and HNF4G expression in these models. A few representative examples are presented to show that pelabresib treatments effectively downregulated HNF1A and HNF4G expression (Figure 5, D and E).

These data suggest that the GI transcriptome expression in prostate cancer, as assessed by HNF score, may serve as a predictive marker of prostate cancer PDX response to pelabresib treatment. We evaluated the growth inhibitory response of an HNF4G/HNF1A^+^ CWR22Pc cell-derived xenograft model to pelabresib treatment in vivo. We found that pelabresib treatment inhibited the growth of the xenografts. The explanted tumors were harvested for RNA and protein extraction at 2 time points (2 days and at end of study). qRT-PCR and immunoblotting studies showed downregulation of HNF1A, HNF4G, and their targets in pelabresib-treated tumors compared with vehicle controls (Supplemental Figure 5, A–C). Taken together, the observed correlations highlight the potential clinical relevance of the GI transcriptome in guiding BETi therapy.

**Figure 6:**
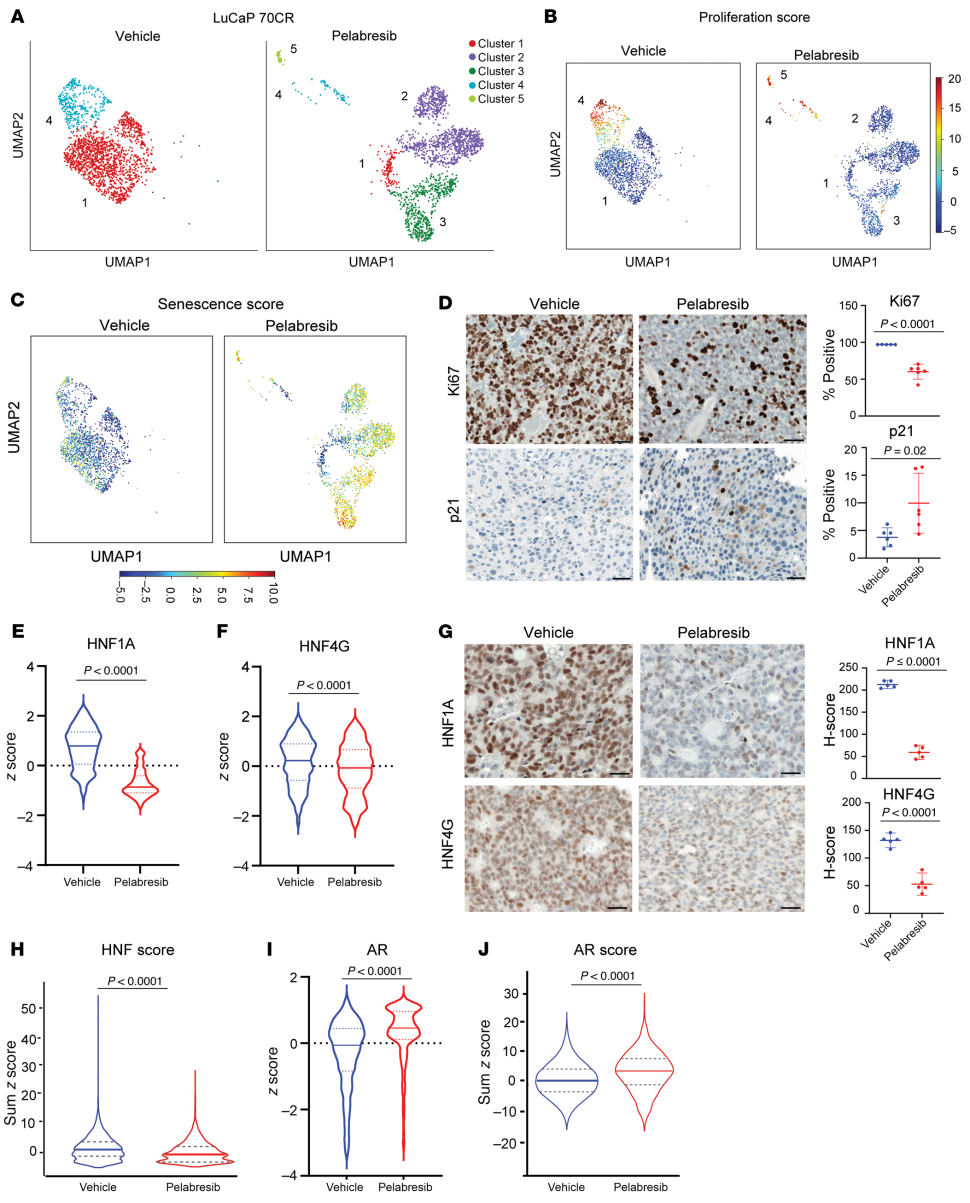
Pelabresib treatment inhibits proliferation and induces senescence in LuCaP 70CR. (**A**) UMAPs of single cells isolated from vehicle- or pelabresib-treated LuCaP 70CR tumors. (**B**) UMAPs depicting proliferation scores of single cells isolated from vehicle- or pelabresib-treated tumors. (**C**) UMAPs depicting senescence scores of single cells isolated from vehicle- or pelabresib-treated tumors. (**D**) Representative IHC staining and quantification of Ki67 and p21 in pelabresib- or vehicle-treated tumors and quantification. Scale bar: 50 μm. *n* = 2. *P* values were obtained from a 2-tailed unpaired *t* test. (**E**) Violin plot of HNF1A expression in single cells obtained from pelabresib- or vehicle-treated tumors. The median is shown by a solid line and the first and third quartiles are shown by dashed lines. The *P* value was obtained from an unpaired *t* test. (**F**) Violin plot of HNF4G expression in single cells obtained from vehicle- or pelabresib-treated tumors. The median is shown by a solid line; the first and third quartiles are shown by dashed lines. The *P* value was obtained from an unpaired *t* test. (**G**) Representative IHC staining and quantification of HNF1A and HNF4G in pelabresib- or vehicle-treated tumors and quantification (*n* = 2). Scale bar: 20 μm. *n* = 2. *P* values were obtained from a 2-tailed unpaired *t* test. (**H**) Violin plot depicting the HNF score in single cells obtained from vehicle- or pelabresib-treated tumors. The median is shown by a solid line; the first and third quartiles are shown by dashed lines. The *P* value was obtained from an unpaired *t* test. (**I**) Violin plot depicting AR expression in single cells obtained from vehicle- or pelabresib-treated tumors. The median is shown by a solid line and the first and third quartiles are shown by dashed lines. The *P* value was obtained from unpaired *t* test. (**J**) Violin plot depicting the AR score in single cells obtained from vehicle- or pelabresib-treated tumors. The median is shown by a solid line and the first and third quartiles are shown by dashed lines. The *P* value was obtained from an unpaired *t* test.

To gain a comprehensive understanding of the molecular changes induced by BET inhibition, we performed single-cell RNA-Seq (scRNA-Seq) on LuCaP 70CR tumors treated with pelabresib for 6 days, using vehicle as control. Tumors were dissociated into single-cell suspension and live cells were obtained using fluorescence-activated cell sorting. We discarded cells with mouse reads and analyzed single transcriptomes from approximately 3,650 single human cells in vehicle- and pelabresib-treated mice (*n* = 2) after quality control and filtering. Dimension reduction using Uniform Manifold Approximation and Projection (UMAP) and Leiden clustering grouped tumor cells into 5 clusters (Figure 6A). In vehicle-treated mice, the majority of tumor cells grouped into cluster 1 and had characteristics of prostate adenocarcinoma, including luminal markers KRT8, KRT18, FOLH1; prostate transcription factors AR, NKX3-1, FOXA1, HOXB13; and GI transcription factors HNF1A, HNF4G, and downstream target like MUC13 (Supplemental Figure 6A). In addition, a fraction of cells grouped into cluster 4, which maintained prostate lineage markers and is additionally characterized by expression of proliferation genes, suggesting this is the proliferative cluster (Figure 6B and Supplemental Figure 6, A and B). Treatment with pelabresib resulted in a decrease in the cell populations in clusters 1 and 4 and an increase or emergence of clusters 2, 3, and 5 (Figure 6A). These 3 clusters all expressed senescence-related genes and exhibited a high senescence score, with the small cluster 5 having high scores for both proliferation and senescence (Figure 6C and Supplemental Figure 6B). We performed Ki67 and p21 IHC staining on pelabresib-treated tumors as markers of proliferation and senescence, respectively. We noticed a decrease in Ki67 staining and an increase in p21 staining in pelabresib-treated tumors compared with vehicle-treated controls (Figure 6D). These data suggest pelabresib treatment inhibits proliferation and induces senescence in these preclinical models.

Pelabresib treatment led to robust decrease in *HNF1A* and modest decrease in *HNF4G* expression assess by scRNA-Seq transcript levels and by IHC (Figure 6, E–G), consistent with in vitro data (Figure 2, A and B, and Figure 4, B and F). To quantify the downstream GI transcriptome, we assigned the previously defined HNF and AR scores to each single cell in both treatment conditions. Pelabresib treatment led to a significant decrease in the HNF score, suggesting an effect on the GI transcriptome (Figure 6H and Supplemental Figure 6C). In contrast, AR expression was not suppressed, and AR score did not decrease with pelabresib treatment (Figure 6, I and J). These findings are consistent with bulk RNA-Seq data on 22Rv1 cells (Figure 2D and Supplemental Figure 2A).

We next performed pseudobulk analysis pooling all single-cell transcriptomic data of each condition to identify differentially expressed genes between pelabresib and vehicle treatment. GSEA analysis of the pseudobulk data showed enrichment of HNF4G targets, as well as cell cycle–related gene sets in pelabresib-downregulated genes. Senescence-related gene sets were enriched in pelabresib-upregulated genes (Supplemental Figure 6D). Collectively, these data indicate that BETi inhibits the GI transcriptome, inhibits proliferation, and induces senescence in GI transcriptome-positive prostate cancer.

### Combination efficacy of enzalutamide and pelabresib in AR-positive CRPC PDX models.

**Figure 7:**
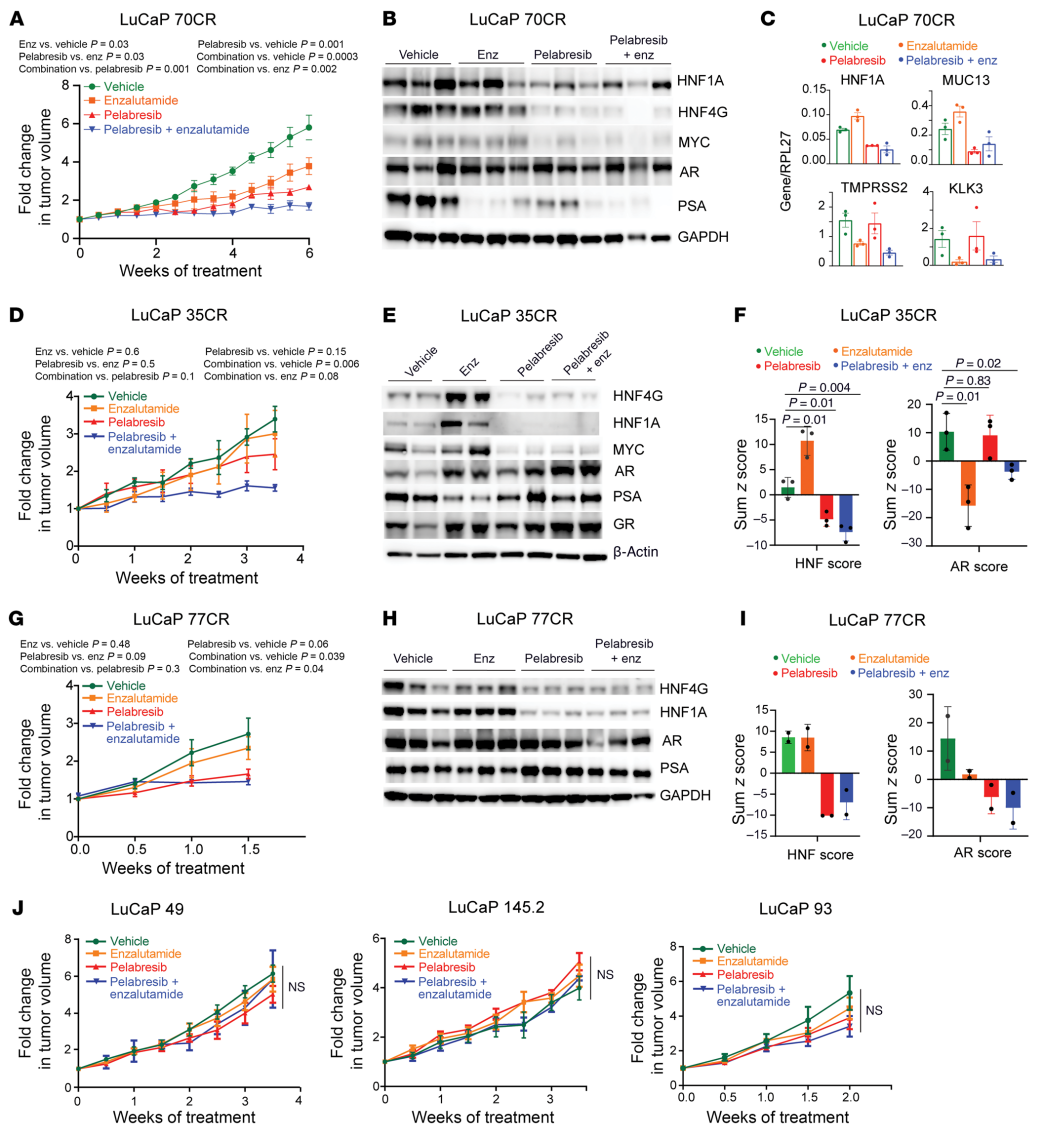
Combination efficacy of enzalutamide and pelabresib in AR-positive CRPC PDX models. (**A**) Treatment response of LuCaP 70CR PDX in SCID mice when treated with vehicle (0.5% methylcellulose/0.2% Tween-80 in sterile water), enzalutamide (50 mg/kg), pelabresib (30 mg/kg), or enzalutamide and pelabresib. Enzalutamide and pelabresib were oral gavaged once and twice a day, respectively (*n* = 5 for all treatments). Treatment was started when tumors reached a volume of approximately 100 mm^3^. Fold change in growth rate over day 0 (start of treatment) is shown. Data are mean ± SEM. *P* values were determined from a 2-tailed unpaired *t* test. (**B**) Immunoblots of 3 representative tumor explants obtained at the end of the experiment shown in **A**. (**C**) qRT-PCR analysis of HNF1A, MUC13, TMPRSS2, and KLK3 mRNA levels in tumors harvested at the end of the study. *n* = 3 for each treatment condition. (**D**) Treatment response of LuCaP 35CR PDX in SCID mice when treated with vehicle, enzalutamide, pelabresib, or enzalutamide and pelabresib. Treatment conditions were the same as described in **A** (*n* = 3 for all treatments). Fold change in growth rate over day 0 (start of treatment) is shown. Data are mean ± SEM. P values were determined from a 2-tailed unpaired *t* test. (**E**) Immunoblots of 2 representative tumors obtained at the end of the study shown in **D**. (**F**) Left panel shows HNF score modulation in LuCaP 35CR tumors treated with different drugs, as shown in **D**. The HNF score was calculated using RNA-Seq gene expression generated from explanted tumors at the end of the study. The right panel shows modulation of AR signaling using the AR score. P values were determined from a 2-tailed unpaired *t* test, *n* = 3. (**G**) Treatment response of LuCaP 77CR PDX in SCID mice when treated with vehicle, enzalutamide, pelabresib, or enzalutamide and pelabresib. Treatment conditions were same as described in **A** (*n* = 3 for all treatments). Fold change in growth rate over day 0 (start of treatment) is shown. Data are mean ± SEM. P values were determined from a 2-tailed unpaired *t* test. (**H**) Immunoblots of 3 representative tumors obtained at the end of the study shown in **G**. (**I**) HNF score (left) and AR score (right) modulation in LuCaP 77CR tumors treated with different drugs as shown in **G**. (**J**) Treatment response of LuCaP 49, LuCaP 145.2, and LuCaP 93 PDXs in SCID mice when treated with vehicle, enzalutamide, pelabresib or enzalutamide and pelabresib. Treatment conditions were same as described in **A**. *n* = 3 for each treatment condition in each PDX line. *P* values were determined from a 2-tailed unpaired *t* test (*n* = 2).

Next, we asked whether BET inhibition could further synergize with AR inhibition in CRPC by targeting a parallel survival pathway of the GI transcriptome. For this we used CRPC PDXs with varied levels of HNF scores—LuCaP 70CR (AR^pos^ HNF^high^), LuCaP 77CR (AR^pos^ HNF^high^), LuCaP 35CR (AR^pos^ HNF^low^), LuCaP 145.2 (NEPC, HNF^neg^), LuCaP 49 (NEPC, HNF^neg^), and LuCaP 93 (NEPC, HNF^neg^)—and treated them with enzalutamide, pelabresib, and a combination of enzalutamide and pelabresib. In LuCaP 70CR, a castration-resistant PDX model, enzalutamide treatment reduced tumor growth rate. Pelabresib treatment induced stronger growth inhibition, and the combination of pelabresib with enzalutamide had the most potent growth inhibitory effects (Figure 7A). Immunoblot analysis performed on tumors collected at the end of the experiment showed a decrease in the protein level of HNF1A in tumors treated with pelabresib alone or in combination with enzalutamide. No significant change in AR protein level was detected with any of the drug treatments (Figure 7B). qRT-PCR analysis performed using RNA extracted from the end-of-study tumors revealed a decrease in selected GI lineage gene transcripts such as HNF1A and MUC13. Enzalutamide treatment decreased AR-target genes’ expression, and the combination treatment decreased both AR and HNF1A/HNF4G target genes’ expression (Figure 7C).

LuCaP 35CR is a castration-resistant PDX model with a low HNF score. LuCaP 35CR tumors were treated with vehicle, enzalutamide, pelabresib, and the combination of pelabresib with enzalutamide for 4 weeks. Tumors showed resistance to enzalutamide treatment. Pelabresib as a single agent had moderate response. However, the combination treatment of pelabresib and enzalutamide significantly reduced tumor growth (Figure 7D). Immunoblot analysis on protein lysates from end-of-study tumors revealed that enzalutamide treatment led to an increase in protein levels of HNF4G and HNF1A (Figure 7E). The increase in HNF1A and HNF4G protein after enzalutamide treatment was also observed by IHC of tumor samples (Supplemental Figure 7A). We have noted similar observations in LNCaP/AR tumors treated with enzalutamide (9). Importantly, the increase in GI gene expression induced by enzalutamide treatment could be effectively inhibited by combining pelabresib with enzalutamide (Figure 7E).

RNA-Seq analysis was performed on LuCaP 35CR tumors to study global transcriptome changes under different treatment conditions. Enzalutamide treatment significantly increased the HNF score. Pelabresib decreased HNF1A expression and the HNF score and, in combination with enzalutamide, reversed the enzalutamide-induced increase in the HNF score (Figure 7F and Supplemental Figure 7B). The AR score decreased with enzalutamide alone and with enzalutamide and pelabresib combination treatment but not with pelabresib treatment alone (Figure 7F and Supplemental Figure 7B).

Similar observations were noted when we performed a short-term treatment study using LuCaP 77CR, a castration-resistant, a high-HNF-score, and AR-positive model. Enzalutamide alone did not cause any significant growth inhibition. In contrast, pelabresib treatment either alone or in combination with enzalutamide significantly reduced the growth of LuCaP 77CR (Figure 7G). Immunoblot analysis of protein lysates prepared from end-of-study tumors revealed that pelabresib treatment, either alone or in combination with enzalutamide, led to a decrease in protein levels of HNF4G and HNF1A. The AR protein level remained unchanged under all treatment conditions (Figure 7H). The effect of pelabresib on HNF1A and HNF4G protein levels was also confirmed by IHC (Supplemental Figure 7C). RNA-Seq analysis performed on end-of-study tumors revealed a decrease in *HNF1A* expression as well as the HNF score with both the pelabresib and the combination treatment; neither *AR* expression nor the AR score altered with any of the treatments (Figure 7I and Supplemental Figure 7D).

Taken together, across different GI transcriptome-expressing CRPC PDX models, consistent pelabresib-mediated growth inhibition was observed. Importantly, global transcriptome analysis consistently showed robust downregulation of HNF1A, HNF4G, and the HNF score with pelabresib treatment in all the PDX models assayed. Furthermore, tumor growth of the GI transcriptome-expression-negative PDX models (LuCaP 49, LuCaP 145.2, and LuCaP 93) was not inhibited by pelabresib treatment (Figure 7J). Taken together, these data suggest a selective growth inhibitory effect of BETis on GI transcriptome-expressing models.

## Discussion

In prostate cancer, lineage plasticity results in extensive reprogramming of the epigenetic landscape, including changes in the cistrome of the master transcription factor FOXA1 (23) or switch to other master transcription factors, such as loss of AR and gain of ASCL1 or NEUROD1 in NEPC (24, 25). We have uncovered that aberrant upregulation of GI master regulators HNF4G and HNF1A alters enhancer landscape and chromatin accessibility conducive to the expression of GI-specific transcriptome in prostate cancer cells. In the present study, we found that high GI transcriptome expression in mCRPC tumors is predictive of poor response to AR-targeted therapies and shorter overall patient survival. Our previous studies have shown that genetic depletion of either HNF1A or HNF4G inhibits GI transcriptome expression. Thus, we reasoned that pharmacological targeting of either HNF1A or HNF4G would be sufficient for therapeutic studies. HNF4G is an orphan nuclear receptor with no well-characterized ligand, and HNF1A is a homeobox domain–containing transcription factor lacking any small molecule binding pocket. Because of expected roadblocks in identifying small molecule inhibitors regulating the activity of these transcription factors, we focused on an alternative approach of inhibiting HNF1A and HNF4G transcription.

Epigenetic therapy has been proposed to target specific lineage states in prostate cancer. Prior studies have suggested that BET, P300/CBP, LSD1, EZH2, and SWI/SNF inhibitors can disrupt AR-mediated transcriptional activity (16, 17, 26–29). Several studies have shown BRD4 is an important cofactor required for AR transcriptional activity (16, 26). One important caveat of these studies is that cells were hormone starved in charcoal-stripped media, and the addition of dihydrotestosterone together with BETi led to severely impaired AR target gene expression compared with the addition of dihydrotestosterone alone. In these studies, and consistent with our observations, the transcription of AR itself was unaffected by BET inhibition. In our studies using 22Rv1 cells, a panel of patient-derived organoids, and a panel of LuCaP PDX models, BRD4 inhibition did not consistently inhibit the AR-regulated transcriptome, though it did inhibit it in LuCaP 77CR.

Despite early excitement of BETis, the clinical data have shown only modest activity. One limitation has been on target toxicity, and early trials of BETis have shown dose-limiting GI and thrombocytopenia toxicities. Encouragingly, a trial of ZEN-3694 was well tolerated at doses at which BET targets showed a 4-fold mean decrease in expression with no dose-limiting toxicities. ZEN-3694 was associated with prolonged disease stabilization in a subset of patients who had ARSI-refractory disease. Although the trial could not precisely define any biomarkers predictive of ZEN-3694 response, patients with low baseline AR signaling in tumors had longer radiographic progression-free survival (rPFS) than patients with high AR signaling (median rPFS = 10.4 vs 4.3 months). These data indicate tumors with a high HNF score show more stem-cell-like features and low AR activity and may benefit by BETi. There were 4 patients with pre-and post-treatment biopsy specimens and RNA-Seq data. In the 1 patient with an elevated HNF signature, treatment with ZEN-3694 significantly inhibited the signature. In terms of AR signaling, approximately 30% of patients experienced an acute increase in PSA upon starting the drug, and this increase was associated with longer PFS, a feature that distinguishes BETi from ARSIs (18). A serum PSA decline of 50% or more (PSA_50_) with ZEN-3694 treatment was seen in only 10% of patients and was not correlated with response to treatment. In another clinical trial using a different BETi GS-5829, only 1 of 31 patients had a PSA_50_ decline (30). These data are consistent with our results and indicate that AR transcriptome is not inhibited by BETi in patients, and AR-independent mechanisms may contribute to BETi response (18).

These data suggest the need for a biomarker-based BETi therapeutic strategy in combination with ARSIs. We now are evaluating IHC staining of HNF4G or HNF1A on pretreatment biopsy specimens as a biomarker of elevated GI signaling in CRPCs. In PDXs, we found an excellent correlation between HNF4G and HNF1A IHC. Our previously published study showed that GI subtype characterized by HNF4G IHC is correlated with GI transcriptome expression in CRPCs (9). Taken together, our findings not only implicate the poor prognosis of GI transcriptome–expressing prostate cancer but also emphasize this subset to be vulnerable to BETi-mediated growth inhibition. Therefore, our studies have important clinical implications, and we propose that a high HNF1A/HNF4G transcriptional activity in CRPC tumors is a biomarker of an aggressive, ARSI-resistant disease that can be managed by treatment with BETis.

## Methods

### Sex as a biological variable.

Our study exclusively examined male mice because the disease modeled is only relevant in male humans.

### Mouse procedures.

CB17 SCID male mice (Charles River) were castrated and, 2 weeks after castration, were subcutaneously implanted with tumor bits of LuCaP 35CR, 70CR, and 77CR. LuCaP 49, 93 and 145.2 were implanted in intact CB17 SCID male mice. When tumors exceeded 100 mm^3^, animals were randomized to control and treatment groups (*n* = 3–6 per group). Treatment with enzalutamide (50 mg/kg, once a day), pelabresib (30 mg/kg, twice daily), enzalutamide and pelabresib combination, or vehicle was begun at a tumor size of 100 mm^3^. Mice were treated until the end of the experiments. Tumor volumes were monitored twice weekly. The research personnel measuring tumors were blinded to the treatment-group assignment of mice.

For CWR22PC xenograft studies, 2.0 × 10^6^ cells resuspended in 100 μL of 1:1 mix of growth medium and Matrigel (BD Biosciences) were subcutaneously injected into CB17-SCID male mice 6–8 weeks old (Taconic). Tumor sizes were measured weekly with calipers starting 4 weeks after xenografting and were calculated using the following formula: tumor volume = (D2 × d2× h2)/6, whereby D, d, and h refer to long diameter, short diameter, and height of the tumor, respectively. Treatment with pelabresib (30 mg/kg) or vehicle was begun at a tumor size of 100 mm^3^. Mice were treated twice daily until the end of the experiments. Two mice from each group were collected 2 days after start of treatment.

### Gene expression analysis.

RNA-Seq was performed by the Memorial Sloan Kettering Cancer Center Integrated Genomics Operation core facility using poly-A capture. The libraries were sequenced on an Illumina NovaSeq 6000 platform with 100 bp paired-end reads to obtain a minimum yield of 40 million reads per sample. The sequence data were processed and mapped to the human reference genome (hg38) or mouse reference genome (mm10) using STAR (RRID:SCR_004463), version 2.3 (31). Gene expression was quantified as transcripts per million using the *edge* R package (32), and log_2_ transformed. GSEA was performed using the JAVA GSEA 2.0 program, using a difference of mean between replicates and gene permutation (33). The gene sets used were the Broad Molecular Signatures Database gene sets v7, c2 (curated gene sets), c5 (gene ontology gene sets), c6 (oncogenic signatures), and c7 (immunologic signatures), as well as custom gene sets we generated.

### Single-cell RNA-Seq.

Subcutaneous PDX tumors were harvested after vehicle or pelabresib treatment (*n* = 2 mice for each condition). The tumors were dissociated into single-cell suspension using the tumor dissociation kit (Miltenyi Biotec, 130-095-929) following the manufacturer’s protocol. Live DAPI-negative, single tumor cells were sorted out by flow cytometry. For each sample, 5,000 cells were directly processed with 10X Genomics Chromium Single Cell 3′ GEM, Library & Gel Bead Kit v3 according to the manufacturer’s specifications. For each sample, 200 million reads were acquired on the NovaSeq platform S4 flow cell. See Supplemental Methods for data analysis.

### Immunohistochemistry.

IHC was performed on an automated Ventana Discovery Ultra Automated IHC Platform. Briefly, FFPE tissue sections were deparaffinized and endogenous peroxidase was inactivated. Antigen retrieval was performed by warming slides to 100°C and incubating for 4 minutes (cell conditioner 1). Sections were then incubated sequentially with the primary antibody overnight, post-primary for 15 minutes, and polymer for 25 minutes, followed by a 10-minute colorimetric development with DAB.

### Analysis of HNF1A and HNF4G IHC in LuCaP tissue microarrays.

IHC was performed in triplicate on tissue microarrays composed of 40 different PDX cores each mounted in triplicate. Staining intensities were quantified using Q-Path software (https://qupath.github.io). Multiple areas were randomly selected in all 3 replicates of each PDX core. Percentage and intensity of nuclear DAB staining were then measured within these regions of interests to obtain a mean H score. The H score was calculated as follows: H score = (1 × number of cells with weak nuclear staining) + (2 × number of cells with moderate nuclear staining) + (3 × number of cells with strong nuclear staining). HNF4G and HNF1A staining are similarly quantified in Figure 5 and Figure 6 (*n* = 2). For Ki67 and p21 staining, instead of H scores, the total number of positively stained cells was determined by selecting different areas of images to include a total of 5,000 cells per treatment condition (*n* = 2).

### Chromatin immunoprecipitation and sequencing.

Chromatin isolation from cell lines and immunoprecipitation were performed following the protocol previously described (9). See Supplemental Methods for details.

### HNF signature and HNF score.

The HNF signature consists of *HNF1A*, *HNF4G*, and their 9 strong, direct downstream targets (*AKR1C3*, *ANG*, *APOH*, *CLRN3*, *GAS2*, *METTL7B*, *MUC13*, *SGK2*, and *UGT2B15*). The 9 candidate genes were chosen if their expression changed with HNF1A/HNF4G knockdown or overexpression in 22Rv1 and LNCaP cells, respectively, and whether their loci showed a direct HNF1A and HNF4G binding (Gene Expression Omnibus [GEO] accession GSE85559 and unpublished data). An HNF score was derived from the summed *z* scores of HNF signature genes expression.

### AR score.

Two previously defined AR signatures (a 10-gene AR signature and an Hieronymus AR Signature) were combined to generate a broader AR signature (11, 12). The AR score is the summed *z* scores of AR signature genes expression.

### Statistics.

All statistical analyses were performed using GraphPad Prism 10 (GraphPad Software). Unless otherwise noted in the figure legends, all data are shown as the mean ± SEM combined with a 2-tailed, unpaired *t* test for statistical comparisons between 2 groups, and a log-rank (Mantel-Cox) test for survival analyses. A *P* value of less than 0.05 was considered statistically significant. All experiments shown were repeated at least twice.

### Study approvals.

The LuCaP PDXs were acquired from rapid autopsies under University of Washington IRB 2341. The MSK-PCa patient-derived organoids were acquired from biopsies under Memorial Sloan Kettering IRB 12-245 or 06-107. All PDX experiments performed at University of Washington were approved under IACUC 3202-01, and all PDX experiments performed at Memorial Sloan Kettering were approved under IACUC 11-12-027.

### Data availability.

Gene Expression Omnibus (GEO) (RRID:SCR_005012) accession numbers of data sets generated are as follows: GSE253805, RNA-Seq expression profile of CRPC PDX LuCaP 77CR with BETi pelabresib and AR inhibitor enzalutamide treatment; GSE253806, scRNA-Seq expression profile of CRPC PDX LuCaP 70CR with BETi pelabresib treatment; GSE254665, RNA-Seq expression profile of CRPC PDX LuCaP 35CR when treated with BETi pelabresib and AR inhibitor enzalutamide; GSE254733, RNA-Seq expression profile of 22Rv1 cells with GFP or HNF4G exogenous expression when treated with BETi ABBV-075; GSE254869, BRD4 ChIP-Seq in 22Rv1 cells exogenously expressing HNF4G or GFP and treated with BETi ABBV-075; and GSE254870, BETi ABBV-075–perturbed pathways in prostate cancer organoid MSK-PCa17.

The data values of all graphs and values behind any reported means in the manuscript are provided in the Supporting Data Values file.

## Author contributions

SS, PC, and YC contributed to the experimental design. SS, DMS, and JY conducted the Western blot and qRT-PCR analyses. WHC conducted IHC. SS conducted and DL analyzed the results of ChIP-Seq, RNA-Seq, and scRNA-Seq. SS also performed cell viability assays. SS, DL, IO, MLR, AT, EK, and YC conducted bioinformatic and biostatistical analyses. E Corey, HMN, JLC, GB, DMS, NAT, and MRP designed and executed the mouse experiments. E Corey, AG, E Campeau, SA, and PT contributed to resources sharing. AG supervised the pathology. SS and YC wrote the manuscript, and all authors reviewed and edited the manuscript.

## Supplementary Material

Supplemental data

Unedited blot and gel images

Supplemental table 1

Supplemental table 2

Supplemental table 3

Supporting data values

## Version 1 06/24/2025

In-Press Preview

## Version 2 08/15/2025

Electronic publication
