# Supplementary material for: BET inhibitors reduce tumor growth in preclinical models of gastrointestinal gene signature–positive castration-resistant prostate cancer
Source: J Clin Invest. 2025 Jun 24;135(16):e180378. doi: 10.1172/JCI180378 (PMC12352905; doi:10.1172/JCI180378)
Supplement: Supplemental data [file jci-135-180378-s029.pdf]

Supplemental Figure 1

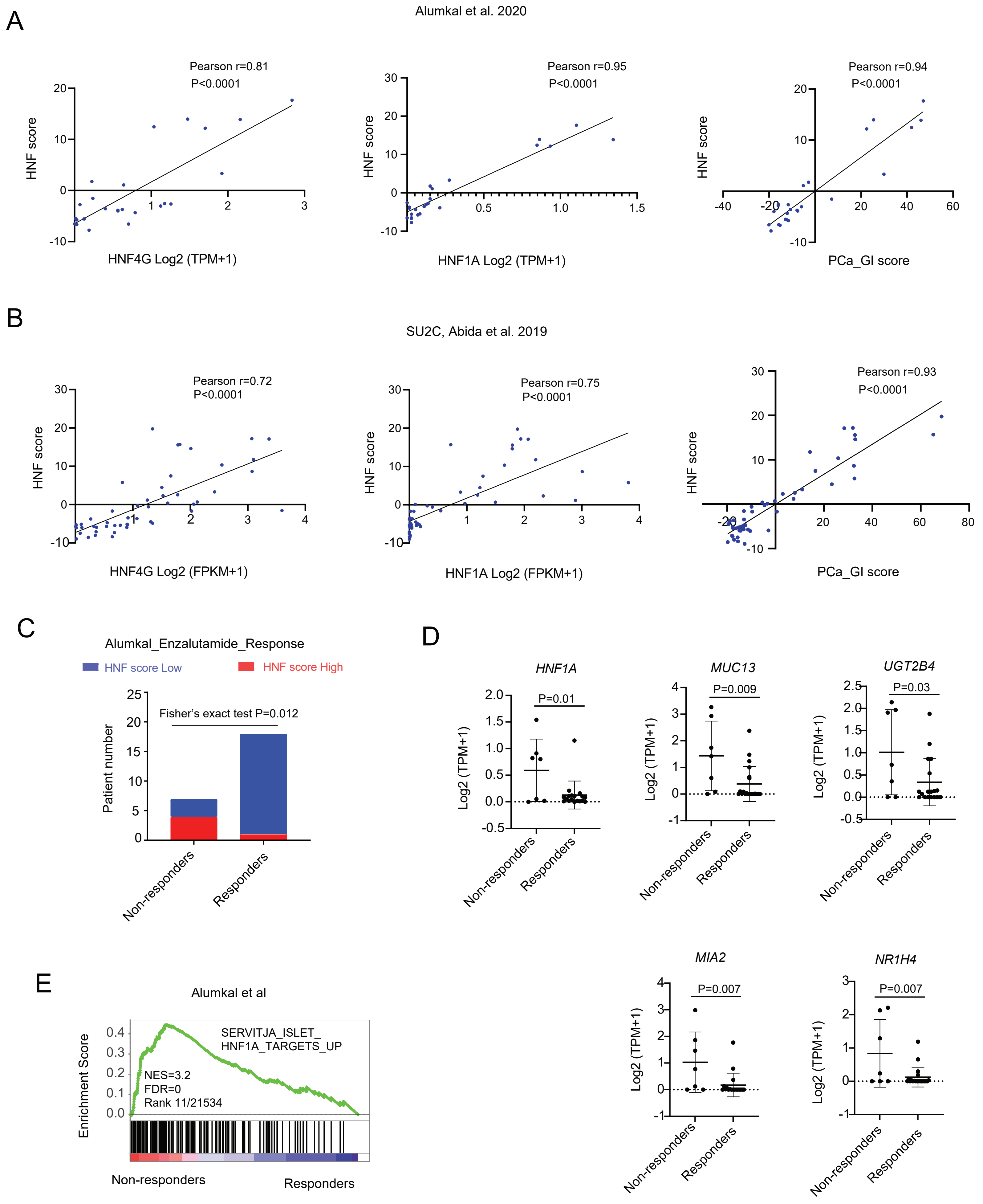

Supplemental Figure 1

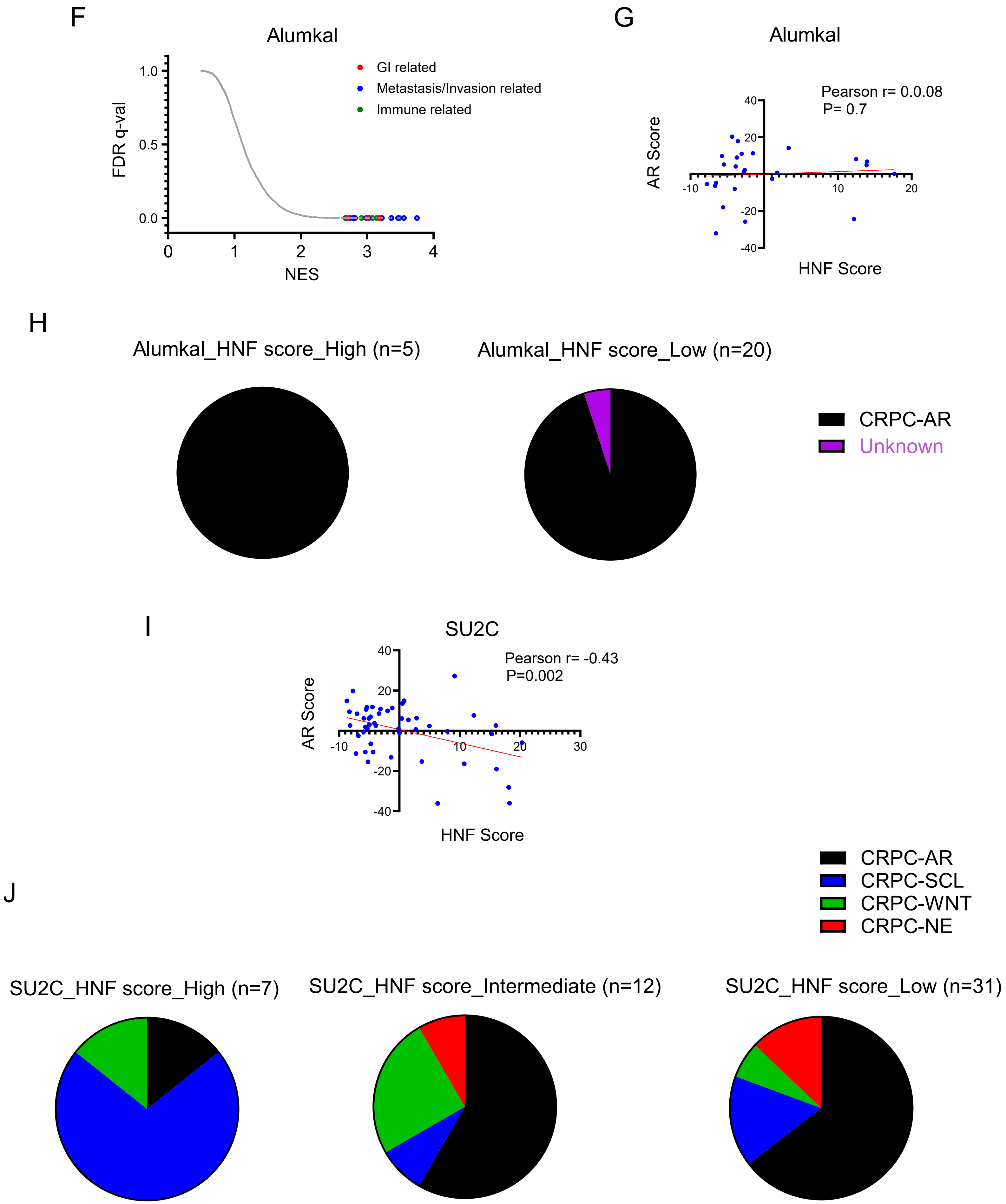

Supplemental Figure 1. Molecular characteristics of HNF score_High tumors

(**A**) Correlation of 11-gene HNF score with HNF4G transcript, HNF1A transcript, and the broader PCa_GI signature sum (Z-score) across all tumors (n=25) for which RNAseq data was available (Alumkal et al. 2020). Pearson's correlation coefficient and p value are indicated on each plot (see Figure 1).

(**B**) Correlation of 11-gene HNF score with HNF4G transcript, HNF1A transcript and the broader PCa_GI signature sum (Z-score) across all Taxane and ARSi naive tumors (n=50) for which RNAseq data was available in the SU2C dataset (Robinson et al. 2015; Abida et al. 2019). Pearson's correlation coefficient and p value are indicated on each plot (see Figure 1).

(**C**) HNF score annotation of tumors of enzalutamide non-responding and responding patients in the Alumkal dataset. Statistical significance is determined using Fisher's exact test.

(**D**) Scatter plots of selected GI-transcriptome genes expression in enzalutamide non-responding and responding tumors. Mean ± SD. Two-tailed unpaired t-test.

(E) GSEA plot of HNF1A target genes in enzalutamide non-responders compared to responders. NES: Normalized enrichment score. FDR: False discovery rate.

(**F**) Global representation of GSEA analysis of RNA-Seq gene expression data comparing non-responders vs responders in Alumkal dataset. The X-axis shows the normalized enrichment score, and the y-axis is the FDR q-value. The GI-related gene sets are indicated in red, metastasis/cellular migration and invasion-related gene sets in blue, and immune-related gene sets in green. NES: Normalized enrichment score. FDR: False discovery rate.

(**G**) Correlation of HNF score with the AR score in the Alumkal data set. Pearson's correlation coefficient and p-value are indicated.

(**H**) Molecular subtype classification of HNF score_High vs HNF score_Low tumors in Alumkal data set.

(**I**) Correlation of HNF score with the AR score in the SU2C data set. Pearson's correlation coefficient and p-value are indicated.

(**J**) Molecular subtype classification of HNF score_High vs HNF score_Low tumors in SU2C data set. CRPC-AR represents the subtype with high AR expression and signaling; CRPC-SCL represents the subtype with Stem Cell-like features; CRPC-WNT represents the subtype with high Wnt signaling, and CRPC-NE represents the subtype with Neuroendocrine features.

Supplemental Figure 2

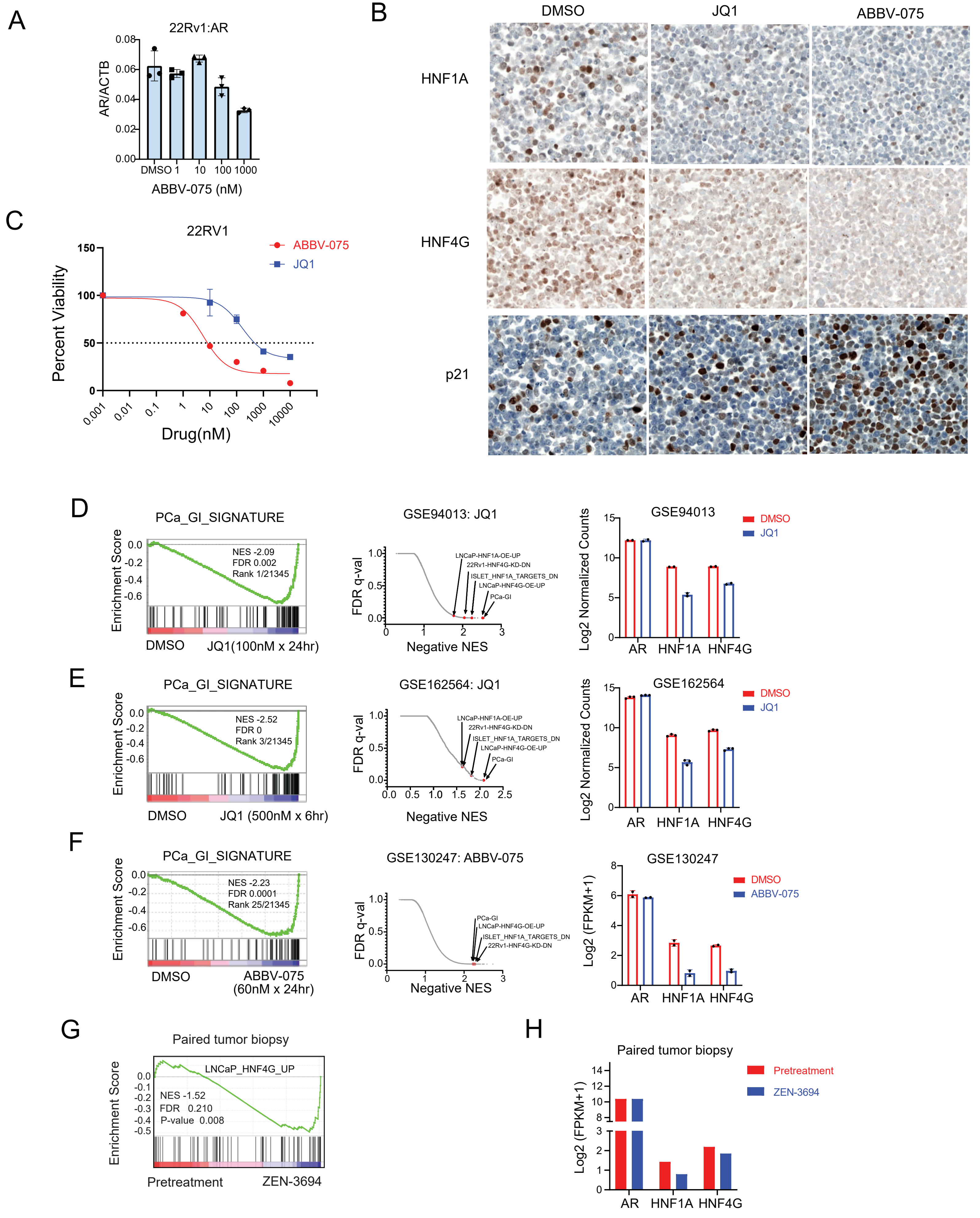

Supplemental Figure 2. BET inhibitors downregulate the GI transcriptome.

(**A**) qRT-PCR of AR expression following 4 hours of treatment with ABBV-075 at indicated doses in 22Rv1 cells.

(**B**) Immunocytochemical staining of 22Rv1 cells treated with JQ1 (250 nM), ABBV-075 (25 nM), and DMSO control for 72 hours. The cells were stained for HNF1A, HNF4G and p21 under these treatment conditions.

(**C**) IC50 curves showing the response of 22Rv1 cells to JQ1 and ABBV-075 treatment. Mean ± SD (n=3).

(**D-F**) GSEA analysis of publicly available RNA-Seq gene expression data sets of 22RV1 cells treated with BET inhibitors JQ1 and ABBV-075. GSEA plots of PCa-GI gene signature is shown in the left panels. Middle panels show the global representation of GSEA for each experiment. X-axis shows the negative normalized enrichment score, and y-axis is the FDR q-value. HNF4G and HNF1A regulated gene sets and the PCa_GI signature gene sets are indicated by arrows. NES: Normalized enrichment score. FDR: False discovery rate. The right panels show expression of AR, HNF1A and HNF4G in each RNA-Seq experiment.

(G) GSEA plots of HNF4G target genes in ZEN-3694 treated tumors compared to pretreated tumor. NES: Normalized enrichment score. FDR: False discovery rate.

(**H**) AR, HNF1A, and HNF4G expression in pre- and post-ZEN-3694 treated patient biopsies.

Supplemental Figure 3

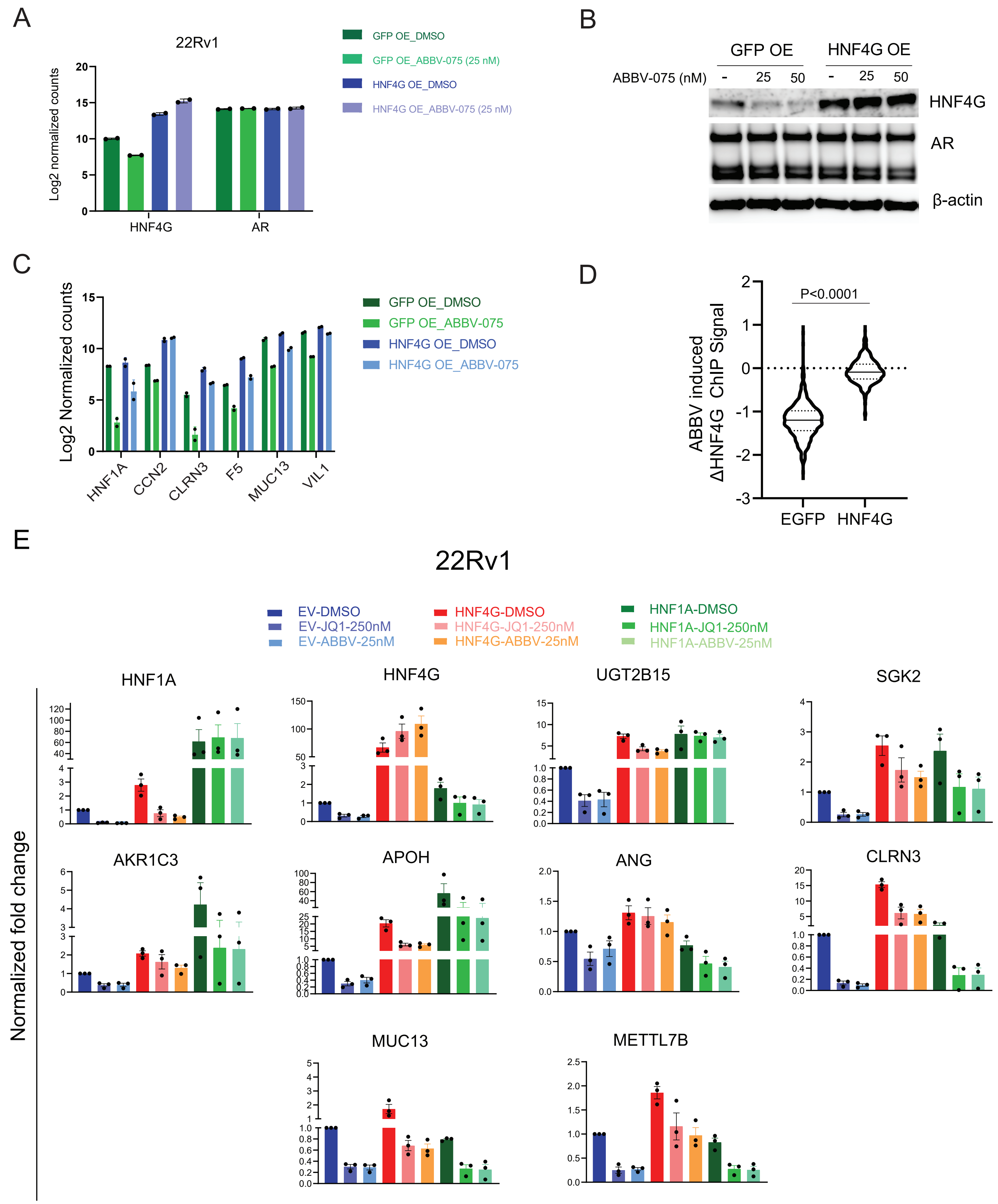

Supplemental Figure 3

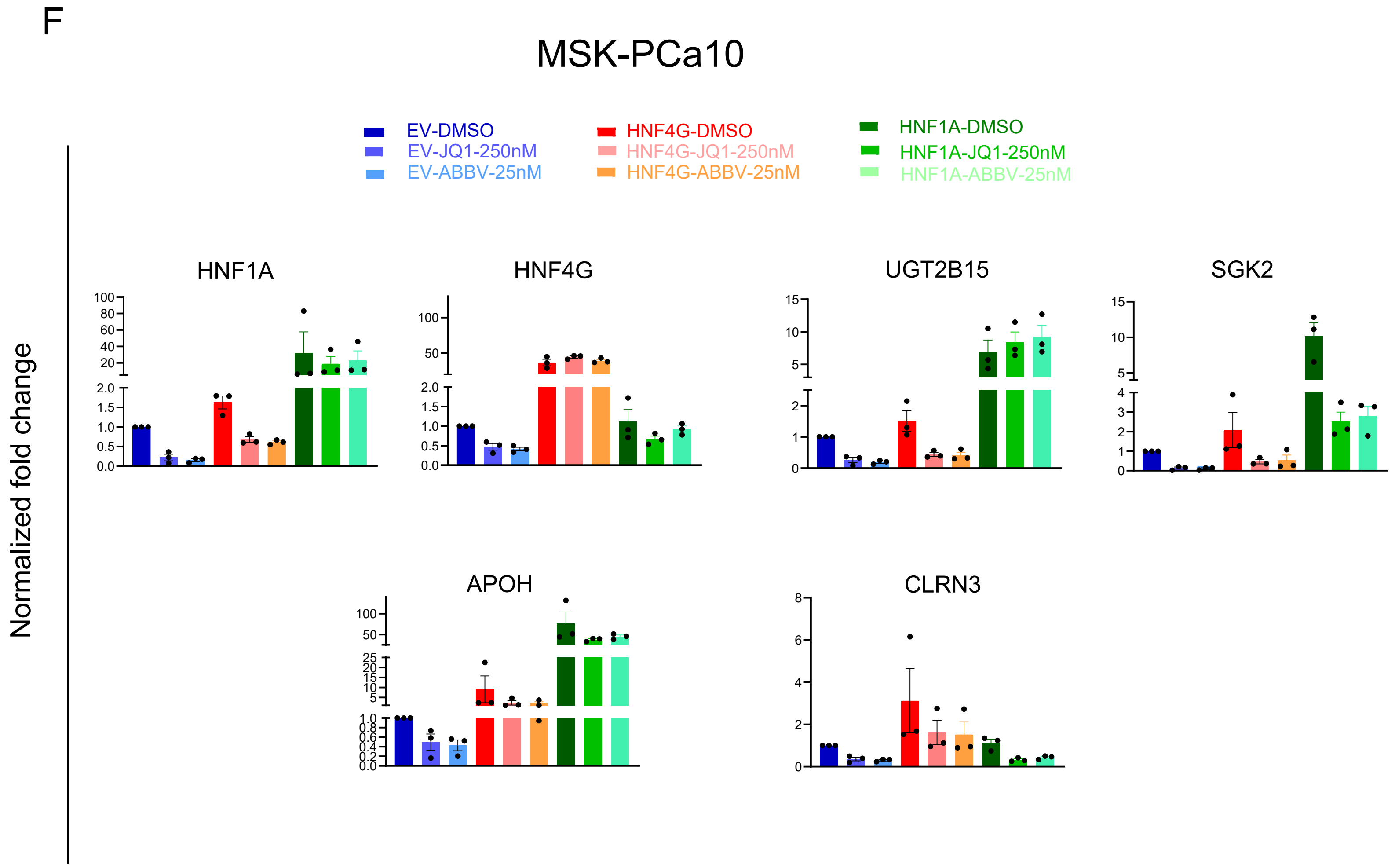

Supplemental Figure 3. HNF1A and HNF4G overexpression can rescue BET inhibitor-mediated downregulation of their target genes.

(**A**) Log2 normalized values of HNF4G and AR expression in 22Rv1 cells overexpressing either GFP or HNF4G from MSCV promoter when treated with DMSO or ABBV-075.
(**B**) Immunoblot depicting HNF4G and AR protein levels in GFP and HNF4G overexpressing 22Rv1 cells treated with ABBV-075 or DMSO for 24 hours.
(**C**) RNA-Seq gene expression values of selected HNF4G target genes after exogenous expression of HNF4G and GFP in 22Rv1cells treated with DMSO or ABBV-075 (25 nM). Data are presented as mean ± SD.
(**D**) A violin plot of the HNF4G peaks that overlapped with BRD4 peaks and their change after ABBV treatment in GFP and HNF4G overexpressing cells. The median is represented by a solid line, while the first and third quartiles are indicated by dashed lines. The p-value was determined using an unpaired t-test.
(**E**) qRT-PCR analysis of 22Rv1 cells overexpressing either empty vector (EV-RFP) control, HNF4G-GFP, HNF1A-RFP and treated with JQ1 (250 nM), ABBV-075 (25 nM), or DMSO control for 24 hours. n=3
(**F**) qRT-PCR analysis of MSK-PCa10 cells overexpressing either empty vector (EV-RFP) control, HNF4G-GFP, HNF1A-RFP and treated with JQ1 (250 nM), ABBV-075 (25 nM), or DMSO control for 24 hours. n=3

Supplemental Figure 4

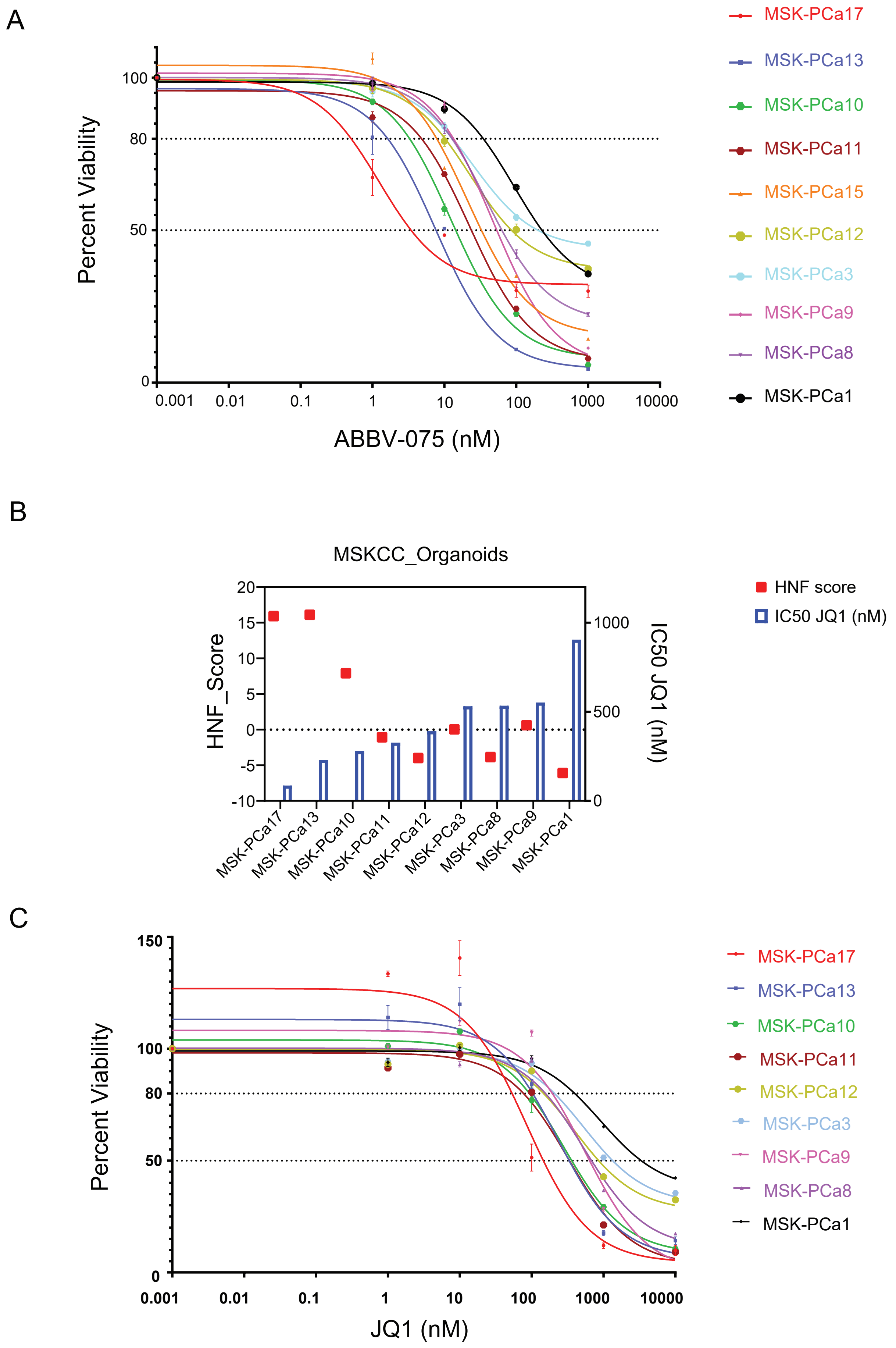

Supplemental Figure 4. IC50 curves of MSK-PCa organoids to ABBV-075 and JQ1 treatment.

(**A**) IC50 curves showing the response of patient-derived organoids to ABBV-075 treatment. Mean ± SD (n=3).

(**B**) IC50 of JQ1 in a panel of patient-derived tumor biopsies grown as organoids. The left Y-axis plots the HNF scores of each organoid and the right Y-axis shows the IC50 values.

(**C**) IC50 curves showing the response of patient-derived organoids to JQ1 treatment. Mean ± SD (n=3).

Supplemental Figure 5

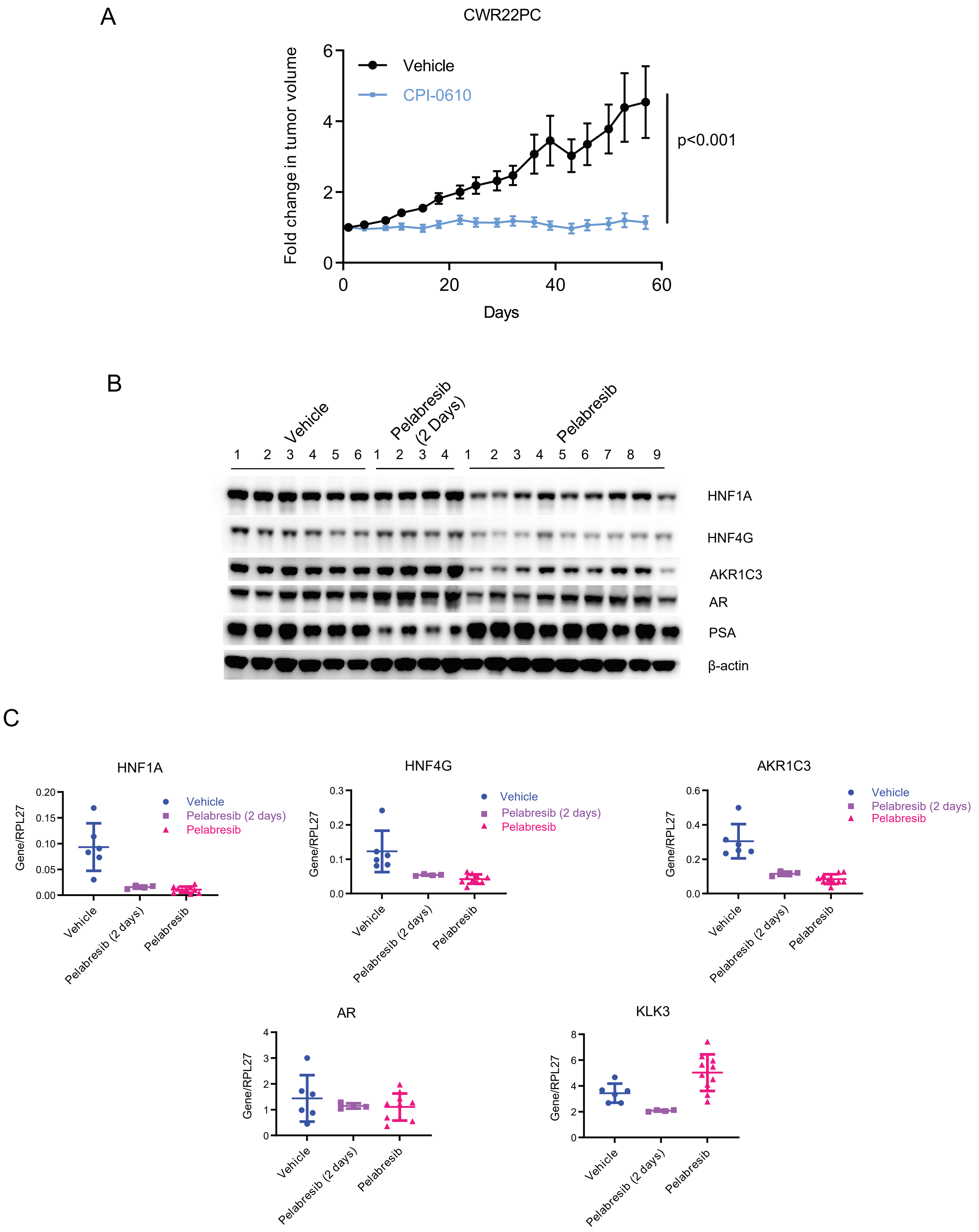

Supplemental Figure 5. In vivo treatment response of HNF1A/HNF4G $^{+}$ CWR22PC cells to pelabresib.

(**A**) Treatment response of CWR22PC cells xenografts in SCID mice when treated with pelabresib (30mg/kg) or vehicle (0.5 % carboxymethyl cellulose) twice a day. For both vehicle and pelabresib, n=10. Treatment was started when tumors reached a volume of approximately 100 mm^3. Fold change in growth rate over day 0 (start of treatment) is shown. Mean ± SEM. Two-tailed unpaired t-test

(**B**) Immunoblotting against selected proteins performed on protein lysate harvested from explanted xenografts at the end of the experiment and 48 hrs post-treatment.

(**C**) qRT-PCR analysis performed on RNA obtained from explanted xenografts at the end of the experiment and 48 hrs post-treatment. Mean ± SD. Two-tailed unpaired t-test.

Supplemental Figure 6

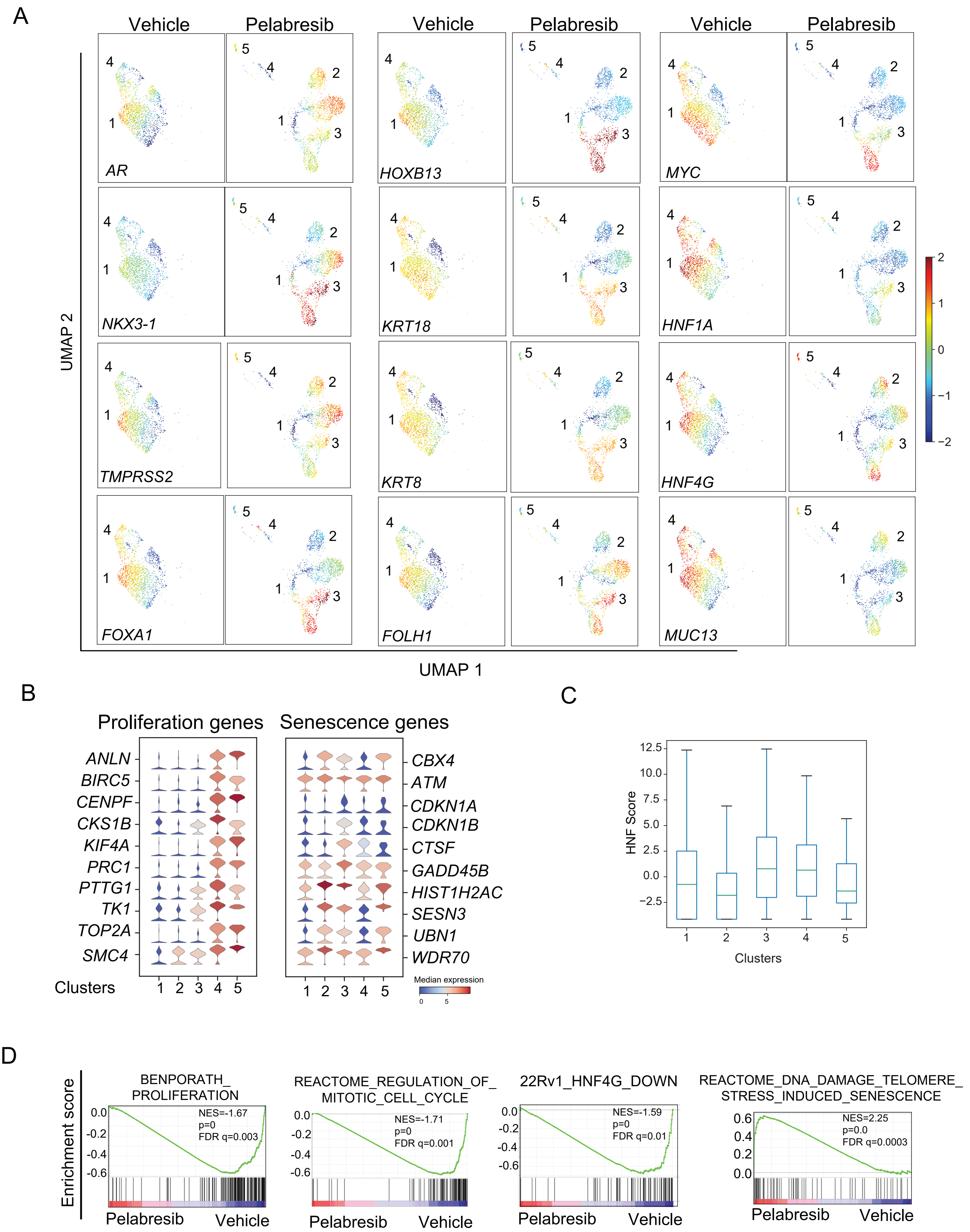

Supplemental Figure 6. Single-cell RNA-seq (scRNA-Seq) of LuCaP 70CR depicting phenotypic and transcriptomic changes with pelabresib treatment.

(**A**) Uniform manifold approximation and projection (UMAP) of scRNA-Seq profiles of selected genes expression in vehicle and pelabresib-treated LuCaP 70CR tumors. Color bar: z-score of log2 counts per ten thousand (log2 [CP10K+1])

(**B**) Violin plots of proliferation and senescence-related marker genes expression across the five clusters observed in UMAP plot of LuCaP 70CR tumors with vehicle and pelabresib treatment. Clusters 1 and 4 are enriched in vehicle-treated tumors while clusters 2, 3, and 5 are enriched with pelabresib treatment. Color in the violin plots indicates the median normalized expression level of genes in each cluster.

(**C**) Box plot depicting HNF scores of each cluster showing median, quartiles, min and max

(**D**) GSEA performed on a ranked list of genes obtained from pseudo-bulk RNA-seq analysis of pooled scRNA-Seq transcriptomics data showing downregulation of proliferative and HNF4G regulated gene sets and enrichment of senescence-related gene sets in pelabresib treated cells. Please see the methods section for details.

Supplemental Figure 7

A

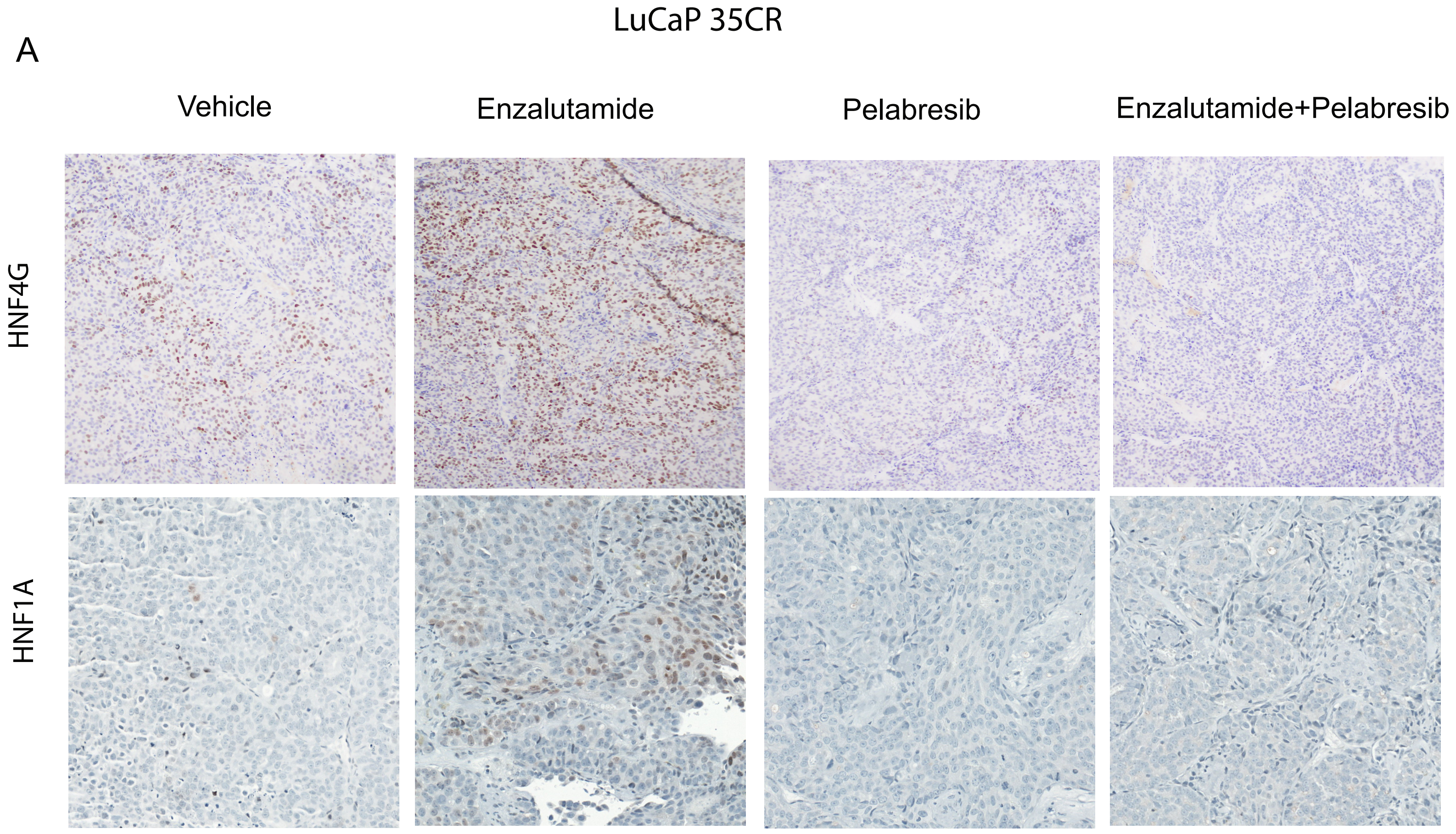

B

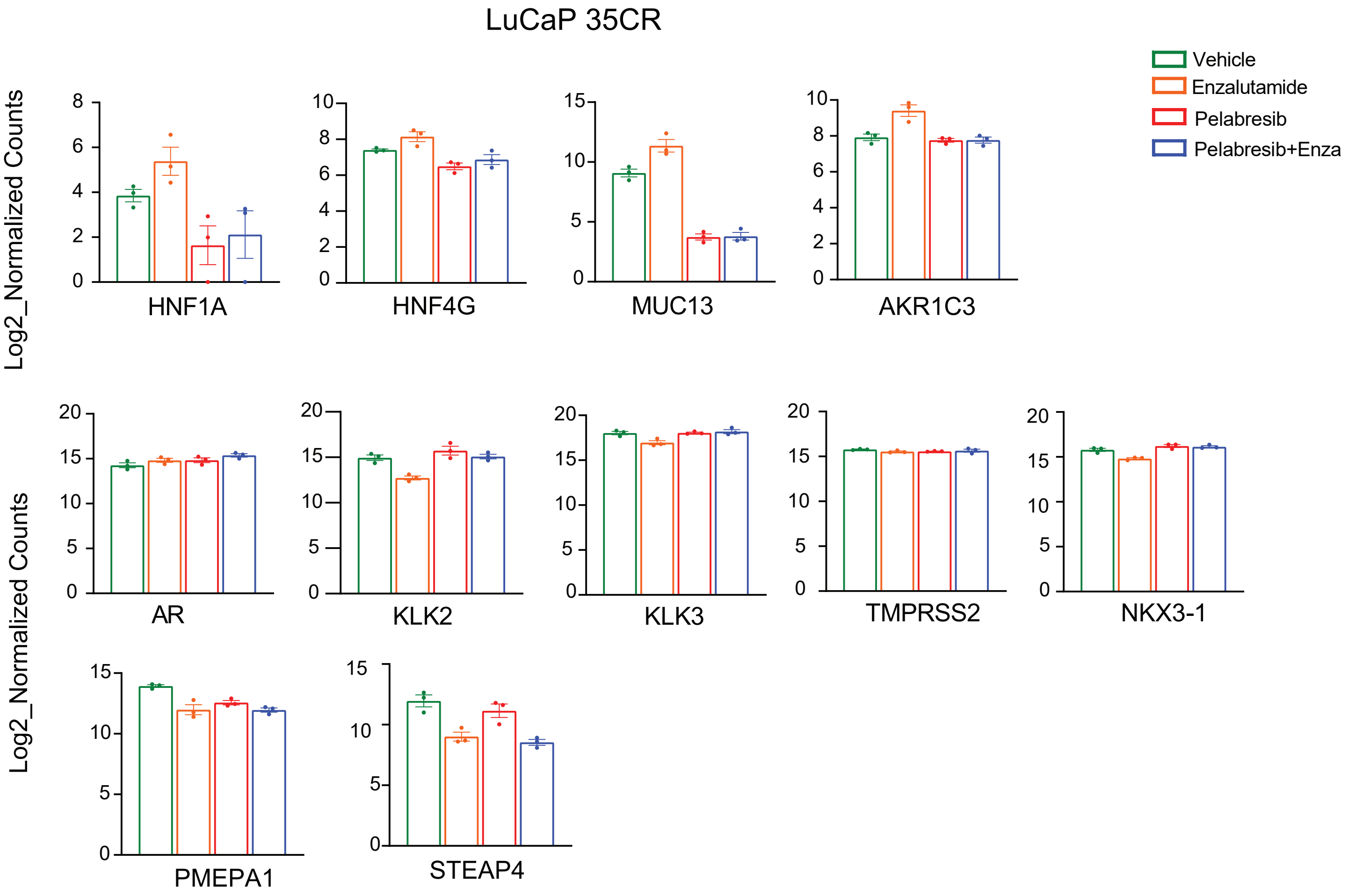

Supplemental Figure 7

C

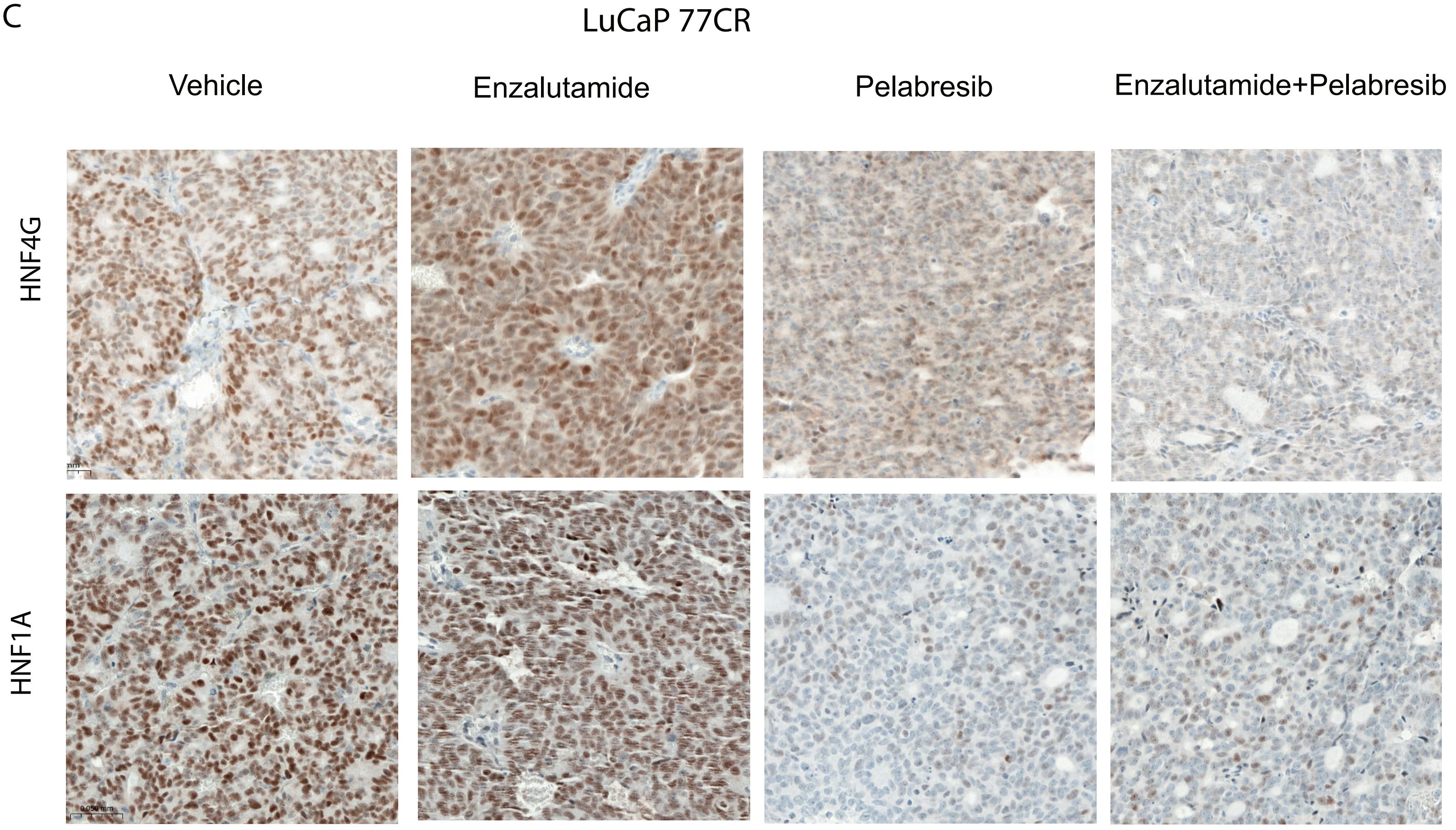

D

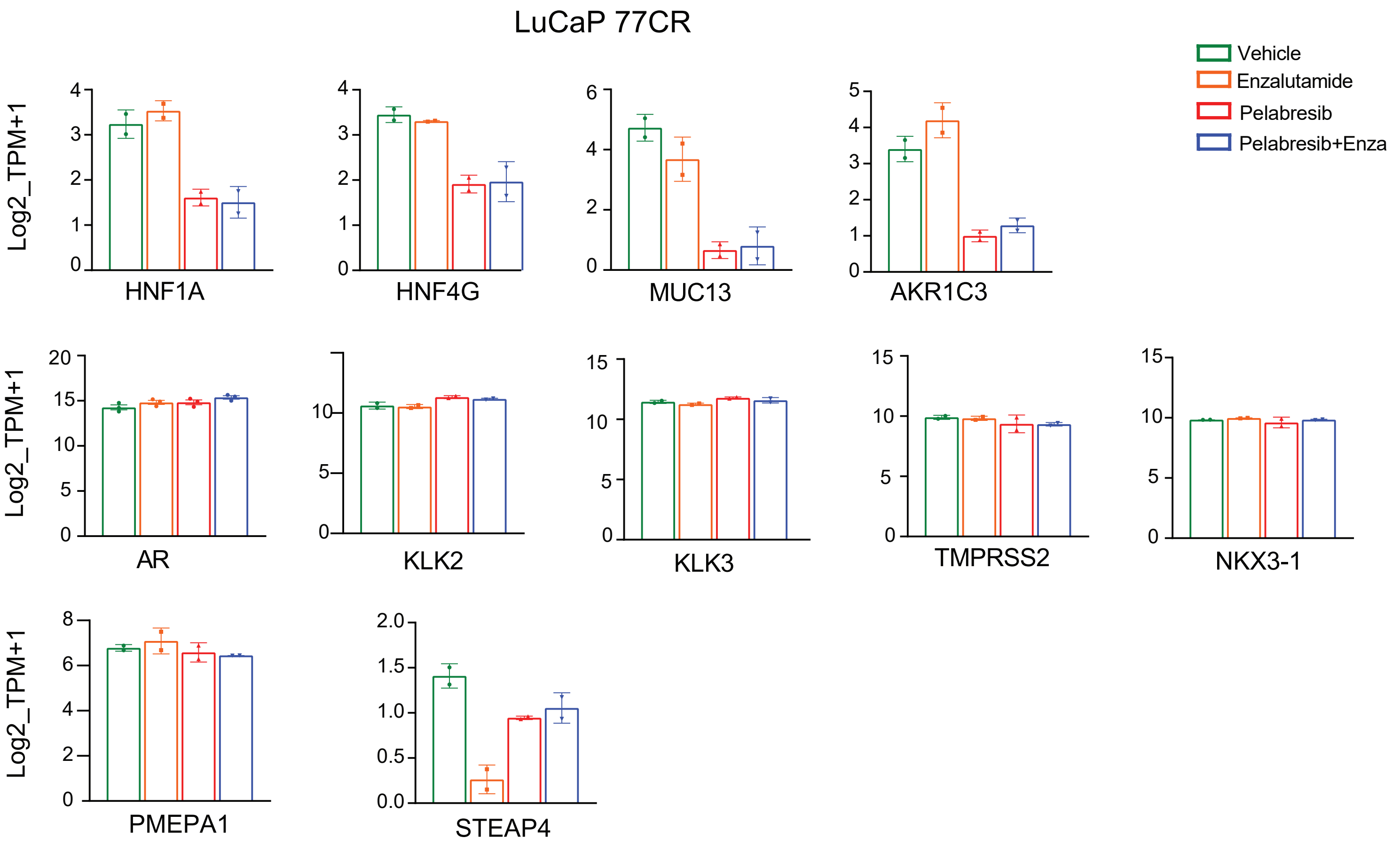

Supplemental Figure 7. Pelabresib treatment downregulates GI genes expression in LuCaP tumors.

(**A**) Representative images of HNF4G and HNF1A immunohistochemical stains performed on LuCaP 35CR tumors under different treatment conditions.

(**B**) RNA-Seq of LuCaP 35CR tumors performed with different treatment conditions showing the expression of selected GI and AR target genes.

(**C**) Representative images of HNF4G and HNF1A immunohistochemical stains performed on LuCaP 77CR tumors under different treatment conditions.

(**D**) RNA-Seq of LuCaP 77CR tumors performed with different treatment conditions showing the expression of selected GI and AR target genes.

Supplemental Methods

Cell lines, Antibodies, and Reagents

22Rv1 (ATCC CRL2505) cell lines was obtained from the American Type Culture Collection (ATCC) and maintained in RPMI supplemented with 10% Fetal bovine serum (Omega), L-glutamine (2 mM), penicillin (100 U/ml), and streptomycin (100 μg/ml). Cell lines were confirmed mycoplasma free by PCR testing.

Antibodies

Antibodies to the following were used for Western blotting: anti-HNF4G (Cell Signaling Technology, Cat# 15742; 1:1000 for Western blotting), anti-HNF1A (Santa Cruj Biotechnology, sc6547X; 1:1000 for Western blotting) anti-HNF1A (Cell Signaling Technology Cat# 89670 (also 89670S), RRID:AB_2728751; 1:1000 for Western blotting), anti-AKR1C3 (Sigma-Aldrich Cat# A6229, RRID:AB_476751; 1:1000 for Western blotting), anti-GAPDH (abmgood, G041; 1:5000 for Western blotting), anti-β-actin (Cell Signaling Technology Cat# 3700; 1:1000 for Western blotting), anti-UGT2B15 (Abcam, ab154864; 1:1000 for Western blotting), anti-MYC (Cell Signaling Technology Cat# 5605, RRID: AB_1903938; 1:2000 for Western blotting), anti-AR (Abcam Cat# ab108341, RRID:AB_10865716); 1:1000 for Western blotting), anti-GR (Cell Signaling Technology Cat# 3660, RRID:AB_11179215; 1:1000 for Western blotting), , anti-PSA (Cell Signaling Technology Cat# 2475, RRID:AB_2797601; 1:1000 for Western blotting), anti-BRD4 (Bethyl laboratories, A301-985A100 for ChIP-seq), anti-HNF4G (Proteintech; 25801-1-AP; for ChIP-Seq),

Antibodies: The following antibodies were used for immunohistochemistry (IHC):

HNF1A: anti-HNF1A antibody ab204306 (Abcam, Abcam Limited, Cambridge, UK) at 1:100 dilution. HNF4G: anti-HNF4G antibody HPA005438 (MilliporeSigma, Billerica, MA, USA) at a

1:50 dilution. P21: anti-p21 Waf1/Cip1 (12D1) 2947 (Cell Signaling Technology, Inc., Danvers, MA) at 1:100 dilution. Ki-67: anti-Ki-67 antibody ab16667 (Abcam, Abcam Limited, Cambridge, UK) at 1:100 dilution.

Immunoblot

Cell lysates were prepared in RIPA buffer containing 1 % SDS and supplemented with proteinase/phosphatase inhibitor. Proteins were resolved on NuPAGE Novex 4–12% Bis-Tris Protein Gels (Life Technologies) and transferred electrophoretically onto a PVDF 0.45 μm membrane (BioRad). Membranes were blocked for 1 hour at room temperature in blocking buffer consisting of 5% milk or 1 % BSA diluted in Tris buffer saline plus 0.1% Tween 20 (TBST) and were incubated overnight at 4 °C with the primary antibodies diluted in the same buffer. After 3 washes of 10 min in TBST, membranes were incubated with secondary antibodies diluted in blocking buffer for 1 hour at room temperature. After 3 washes of 10 minutes in TBST, Enhanced Chemiluminiscence (ECL) was performed using ECL kit (Thermo Scientific, 80196).

Immunocytochemical staining of 22Rv1 cells.

22Rv1 cells were treated with 250nM JQ1 and 25 nM ABBV-075 for 72 hours, the cells were harvested, and cell pellets were then processed by the MSK Molecular Cytogenetics core facility for paraffin embedding. The HNF1A, HNF4G, and p21 staining were then performed on FFPE sections as described in the Immunohistochemistry section above.

Patient Subtype Assignment using EPIC:

We used RNA-seq data from patient samples to assign a CRPC subtype to each patient. We did this using the EPIC deconvolution package, which involves several steps. First, signature expression profiles are created for each CRPC subtype. This is done by aggregating the expression profiles in cell lines/organoids of the different CRPC subtypes in subtype-specific signature genes

described in (1).There are 93 signature genes per CRPC subtype. This results in a signature matrix with the reference expression profile of each CRPC subtype in the signature genes. Next, the CRPC signature gene expression of patient samples is regressed onto the signature matrix to estimate the fractional contribution of each CRPC subtype to the patient's expression profile. Each patient was assigned the subtype with the largest contribution if it was >=0.5. If no subtype satisfied this criterion, we assigned "unknown".

RNA isolation and qRT-PCR

To isolate RNA from cell lines, E.Z.N.A total RNA kit (Omega) was used. To isolate RNA from xenograft tumor explants, the tumor samples were ground in 1 ml Trizol (Invitrogen) using a PowerGen homogenizer (Fisher Scientific), followed by the addition of 200 µL chloroform. The samples were then centrifuged at 10,000 g for 15 minutes. The upper phase was mixed with an equal volume of 70% ethanol, and the RNA was further purified using the E.Z.N.A total RNA kit (Omega).

For qRT-PCR, RNA was reverse transcribed using the High-Capacity cDNA Reverse Transcription Kit (ABI). Power SYBR Master Mix (ABI) was used to run PCR on a ViiA7 Real Time PCR System (Life Technologies).

Cell viability assays

Cells were plated onto 96-well plates in their respective culture medium and incubated at 37 °C in an atmosphere of 5% CO2. After overnight incubation, a serial dilution of ABBV-075 and JQ1 was prepared and added to the plate. The cells were further incubated for 5 days, and the CellTiter-Glo assay (Promega) was then performed according to the manufacturer's instructions to determine cell proliferation. The luminescence signal from each well was acquired using the

GLOMAX 96 microplate Luminometer (Promega), and the data were analyzed using GraphPad Prism software (RRID:SCR_002798).

ChIP-Sequencing

Briefly, chromatin was isolated from 22Rv1 cells expressing HNF4G or GFP and treated with DMSO or ABBV-075. BRD4 ChIP were performed using the anti-BRD4 (Bethyl laboratories, A301-985A100; 10 µg for ChIP-seq). HNF4G ChIP were performed using rabbit anti-HNF4G (Proteintech; 25801-1-AP; 10 µg for ChIP-Seq). Input DNA was also sequenced. Next-generation sequencing was performed on an Illumina NovaSeq 6000 platform with 100 bp paired-end reads. Reads were aligned to the human genome (GRCh38) using the Bowtie2 alignment software (2, 3). Peaks that overlapped with known blacklisted regions (4) were excluded using Homer mergePeaks (5). Duplicate reads were eliminated for subsequent analysis. Peak calling was performed using MACS 2.1 "callpeak" function comparing immunoprecipitated chromatin with input chromatin, using standard parameters and a q-value cutoff of 10^{-2} (6). To generate a set of BRD4 peaks in all conditions, we used Homer "mergePeaks -d given" of peaks with HNF4G and GFP overexpression with DMSO and ABBV-075 treatment (5). We subsequently merged BRD4 peaks with AR and HNF4G peaks from our prior work (GSM2277166 and GSM2277158) to generate a set of all peaks. To generate counts per peak, we used featureCounts (7). We normalized the BRD4 ChIP-seq counts between HNF4G and GFP infected cells to have the same median overall peak counts. We next separated the peaks into promoter and non-promoter (i.e. enhancer) using Homer annotate.

We separately visualized 3 peak sets: 1) Non-promoter sites containing top 1,000 AR peaks that overlap with BRD4 peaks, 2) Non-promoter sites containing top 1,000 HNF4G peaks that overlap with BRD4 peaks, and 3) BRD4 enhancer peaks that do not overlap with AR or HNF4G. The

ChIP-seq profiles presented were generated using Integrated Genome Viewer (IGV) software of bigWig format files, generated using the "bamCoverage" tool from deepTool2 (8).

Single cell RNA-sequencing analysis

Reads obtained from the 10x Genomics scRNAseq platform were mapped to human and mouse combined genome (GRCh38 + mm10) using Cell Ranger (10X Genomics). Human and mouse cells were separated based on the ratio of mapped reads to each genome. Cells were clustered as human or mouse if they had more than 75% mapped reads with human or mouse origin. The raw sequencing fastq data of human and mouse cells were separated using the cell barcodes. The separated human and mouse fastq data were then mapped to human or mouse specific genomes separately using Cell Ranger and downstream analysis was performed separately.

True cells were distinguished from empty droplets using scCB2 package (9). The levels of mitochondrial reads and numbers of unique molecular identifiers (UMIs) were similar among the samples, which indicates that there were no systematic biases in the libraries prepared from mice with different treatment conditions. Cells were removed if they had less than 2000 total counts, more than 60,000 total counts, less than 100 total genes, or greater than 20% mitochondrial reads. Genes detected in less than 20 cells and all mitochondrial genes were removed for subsequent analyses. Putative doublets were removed using the Doublet Detection package (10). The average gene detection in each cell type was similar among the samples. Combining human cells in the entire cohort of pelabresib and vehicle groups yielded a filtered count matrix of 17,475 cells by 23,050 genes, with a median of 12,517 counts and a median of 3,711 genes per cell. The count matrix was then normalized by counts per ten thousand (CP10K), and log(X+1) transformed for analysis of the combined dataset. The top 2000 highly variable genes were found using SCANPY (version 1.6.1) (11). Principal Component Analysis (PCA) was performed on the 2000 most

variable genes with the top 50 principal components (PCs) retained with 25% variance explained. To visualize single cells of the global atlas, we used Uniform Manifold Approximation and Projection for Dimension Reduction (UMAP) (12). We then performed Leiden clustering and found 5 clusters (13). Marker genes for each cluster were found with scanpy.tl.rank_genes_groups. Cell types were determined using a combination of marker genes identified from the literature and gene ontology for cell types using the web-based tool Panglao DB (14). Hierarchical clustering and heat-map generation were performed for single cells based on log-normalized and scaled expression values of marker genes curated from literature or identified as highly differentially expressed.

Differentially expressed genes between different clusters were found using MAST package (15), which were shown in heatmap. The log FC of MAST output was used for the ranked gene list in GSEA analysis (16) .

Gene imputation was performed using MAGIC (Markov affinity-based graph imputation of cells) package, and imputed gene expression were used in theviolin plots, UMAPS, and the heatmap in Figures 5E-F, 5H, S5A and S5C (17).

Differentially expressed genes between pelabresib and vehicle treated samples were found using 'FindMarkers' function of Seurat package (18). The logFC of 'FindMarkers' output was used for the ranked gene list in GSEA analysis (16).

REFERENCES:

1. Tang F, Xu D, Wang S, Wong CK, Martinez-Fundichely A, Lee CJ, et al. Chromatin profiles classify castration-resistant prostate cancers suggesting therapeutic targets. *Science.* 2022;376(6596):eabe1505.
2. Langmead B, Trapnell C, Pop M, and Salzberg SL. Ultrafast and memory-efficient alignment of short DNA sequences to the human genome. *Genome Biol.* 2009;10(3):R25.
3. Langmead B, and Salzberg SL. Fast gapped-read alignment with Bowtie 2. *Nat Methods.* 2012;9(4):357-9.
4. Amemiya HM, Kundaje A, and Boyle AP. The ENCODE Blacklist: Identification of Problematic Regions of the Genome. *Sci Rep.* 2019;9(1):9354.
5. Heinz S, Benner C, Spann N, Bertolino E, Lin YC, Laslo P, et al. Simple combinations of lineage-determining transcription factors prime cis-regulatory elements required for macrophage and B cell identities. *Mol Cell.* 2010;38(4):576-89.
6. Zhang Y, Liu T, Meyer CA, Eeckhoute J, Johnson DS, Bernstein BE, et al. Model-based analysis of ChIP-Seq (MACS). *Genome Biol.* 2008;9(9):R137.
7. Liao Y, Smyth GK, and Shi W. featureCounts: an efficient general purpose program for assigning sequence reads to genomic features. *Bioinformatics.* 2014;30(7):923-30.
8. Ramirez F, Ryan DP, Gruning B, Bhardwaj V, Kilpert F, Richter AS, et al. deepTools2: a next generation web server for deep-sequencing data analysis. *Nucleic Acids Res.* 2016;44(W1):W160-5.
9. Ni Z, Chen S, Brown J, and Kendziorski C. CB2 improves power of cell detection in droplet-based single-cell RNA sequencing data. *Genome Biol.* 2020;21(1):137.
10. Xi NM, and Li JJ. Protocol for executing and benchmarking eight computational doublet-detection methods in single-cell RNA sequencing data analysis. *STAR Protoc.* 2021;2(3):100699.
11. Wolf FA, Angerer P, and Theis FJ. SCANPY: large-scale single-cell gene expression data analysis. *Genome Biol.* 2018;19(1):15.
12. Becht E, McInnes L, Healy J, Dutertre CA, Kwok IWH, Ng LG, et al. Dimensionality reduction for visualizing single-cell data using UMAP. *Nat Biotechnol.* 2018.
13. Traag VA, Waltman L, and van Eck NJ. From Louvain to Leiden: guaranteeing well-connected communities. *Sci Rep.* 2019;9(1):5233.
14. Franzen O, Gan LM, and Bjorkegren JLM. PanglaoDB: a web server for exploration of mouse and human single-cell RNA sequencing data. *Database (Oxford).* 2019;2019.
15. Finak G, McDavid A, Yajima M, Deng J, Gersuk V, Shalek AK, et al. MAST: a flexible statistical framework for assessing transcriptional changes and characterizing heterogeneity in single-cell RNA sequencing data. *Genome Biol.* 2015;16:278.
16. Subramanian A, Tamayo P, Mootha VK, Mukherjee S, Ebert BL, Gillette MA, et al. Gene set enrichment analysis: a knowledge-based approach for interpreting genome-wide expression profiles. *Proc Natl Acad Sci U S A.* 2005;102(43):15545-50.
17. van Dijk D, Sharma R, Nainys J, Yim K, Kathail P, Carr AJ, et al. Recovering Gene Interactions from Single-Cell Data Using Data Diffusion. *Cell.* 2018;174(3):716-29 e27.
18. Hao Y, Hao S, Andersen-Nissen E, Mauck WM, 3rd, Zheng S, Butler A, et al. Integrated analysis of multimodal single-cell data. *Cell.* 2021;184(13):3573-87 e29.
