# Supplementary material for: BET inhibitors reduce tumor growth in preclinical models of gastrointestinal gene signature–positive castration-resistant prostate cancer
Source: J Clin Invest. 2025 Jun 24;135(16):e180378. doi: 10.1172/JCI180378 (PMC12352905; doi:10.1172/JCI180378)

Figure 2C

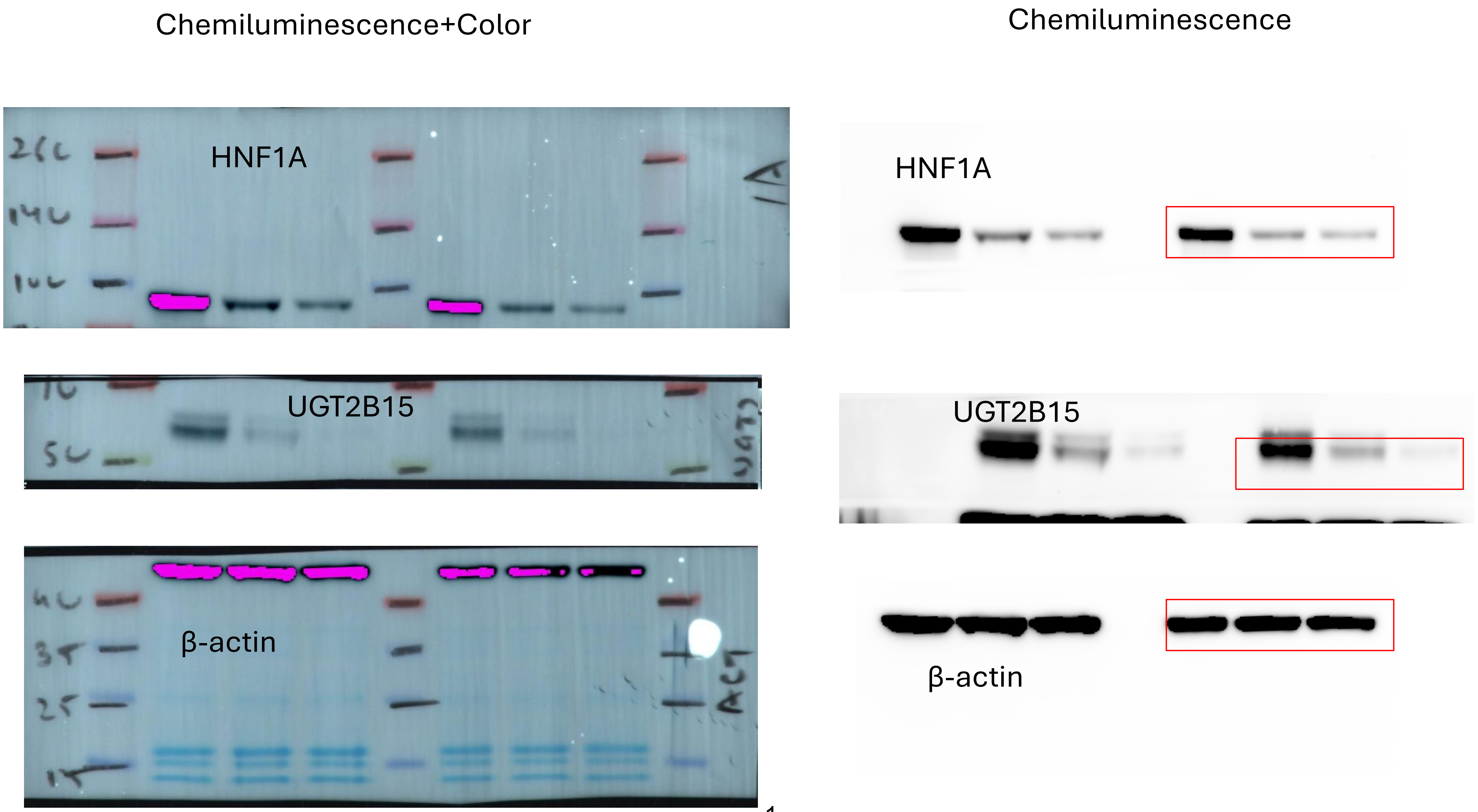
Chemiluminescence+Color
Chemiluminescence
HNF1A
UGT2B15
β-actin
HNF1A
UGT2B15
β-actin

Figure 2C

Chemiluminescence+Color

Chemiluminescence

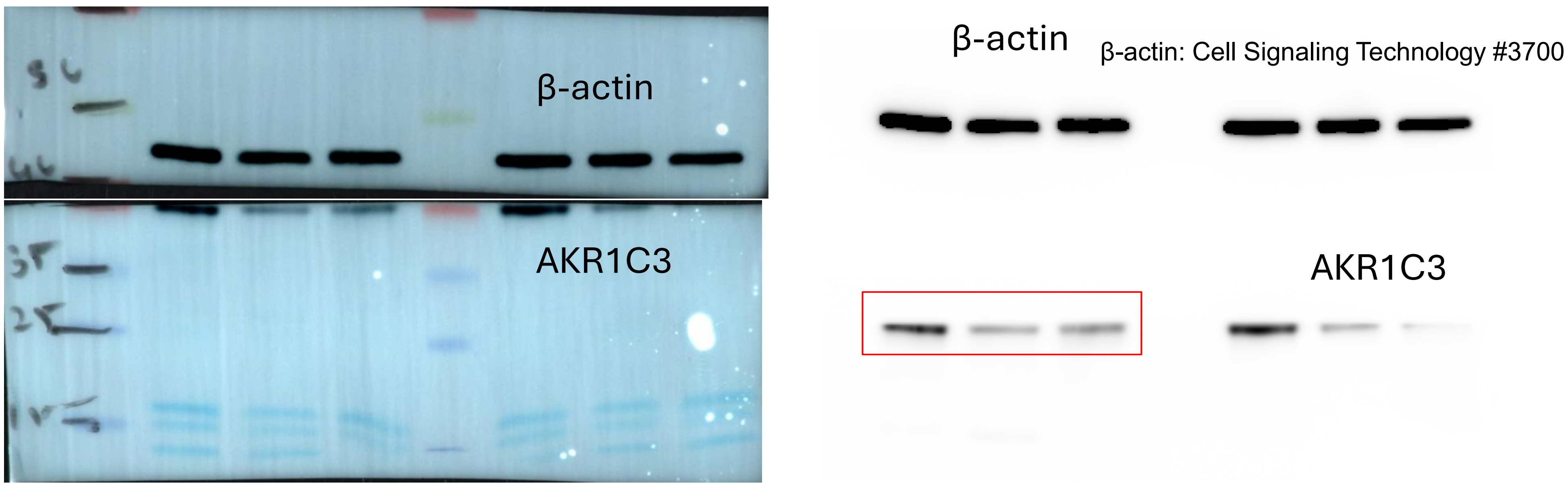

Figure 2C

Chemiluminescence+Color

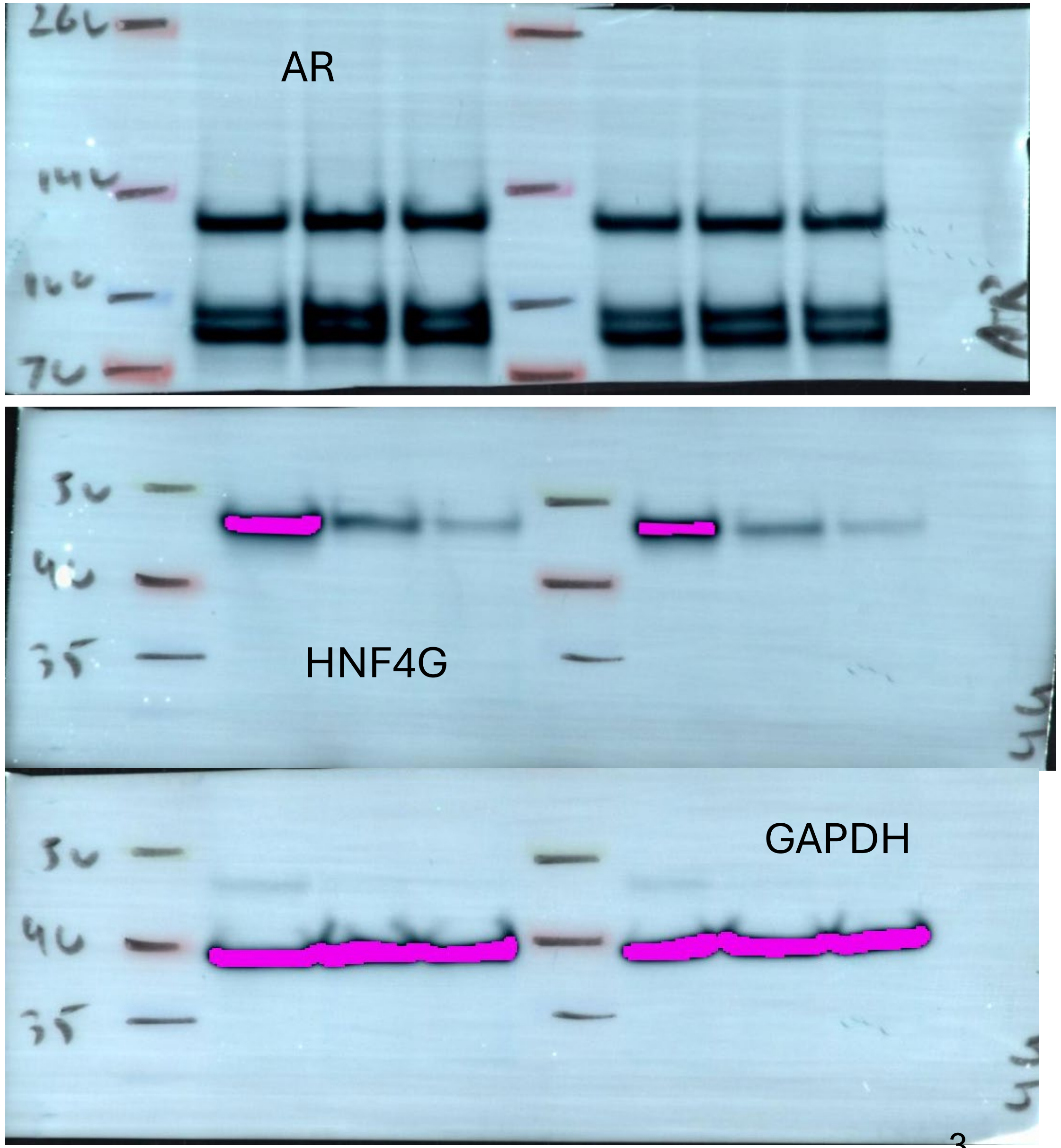

Chemiluminescence

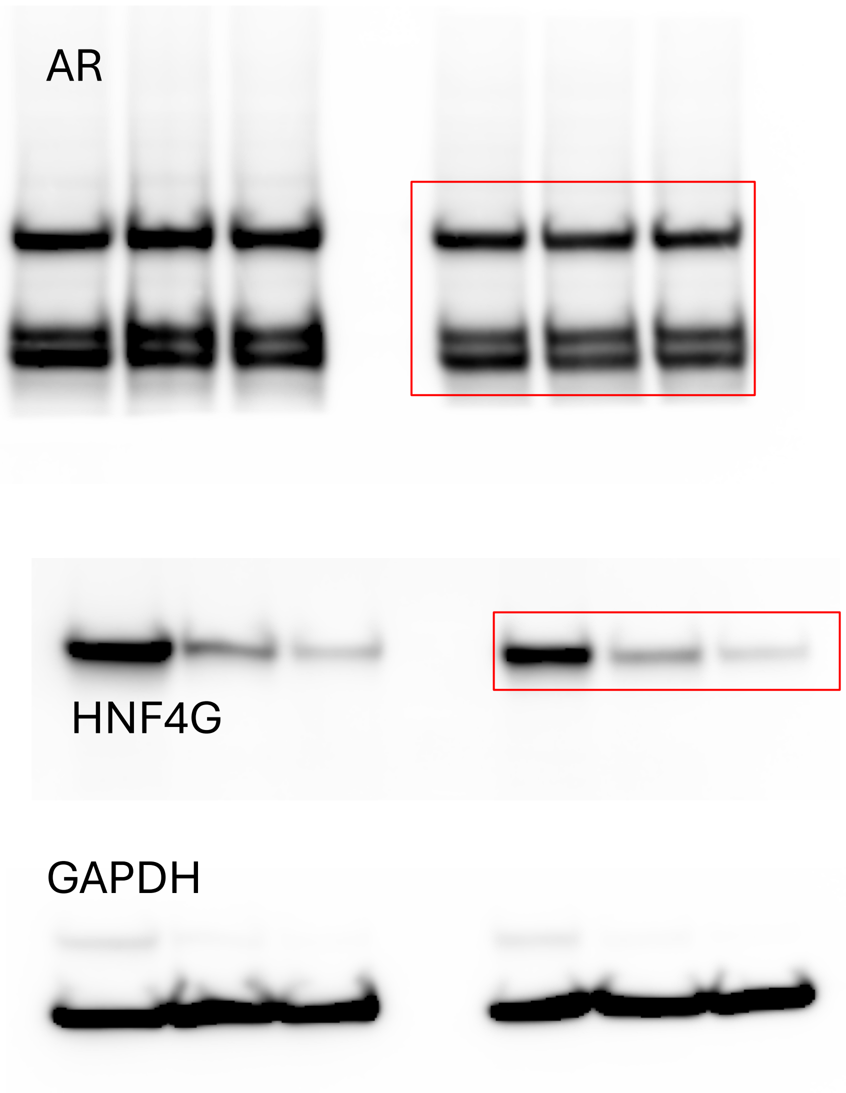

Figure S3B

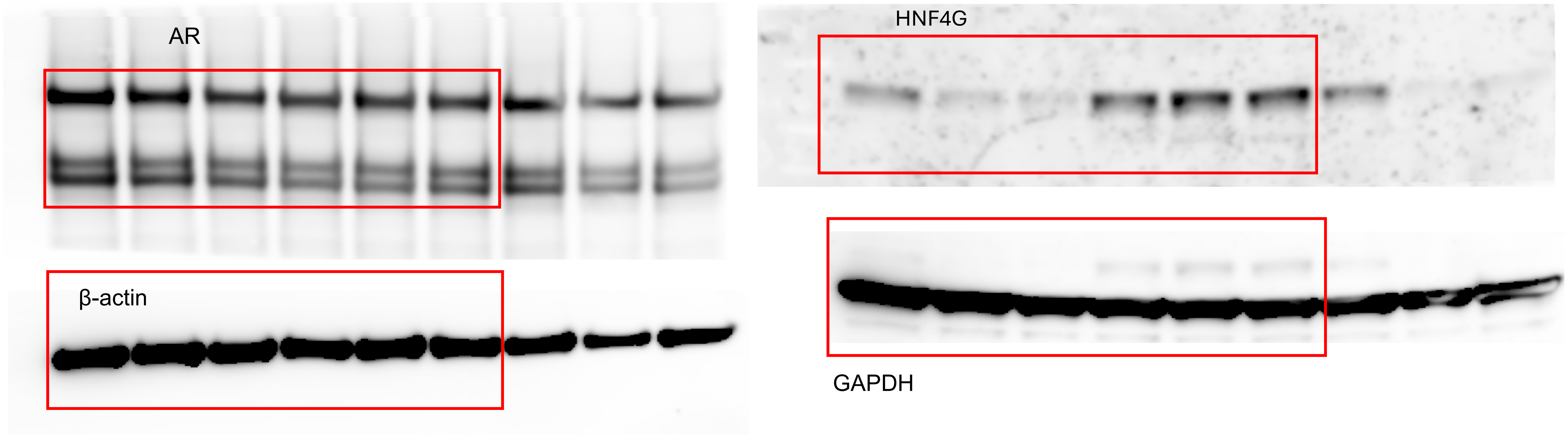

Figure S5B

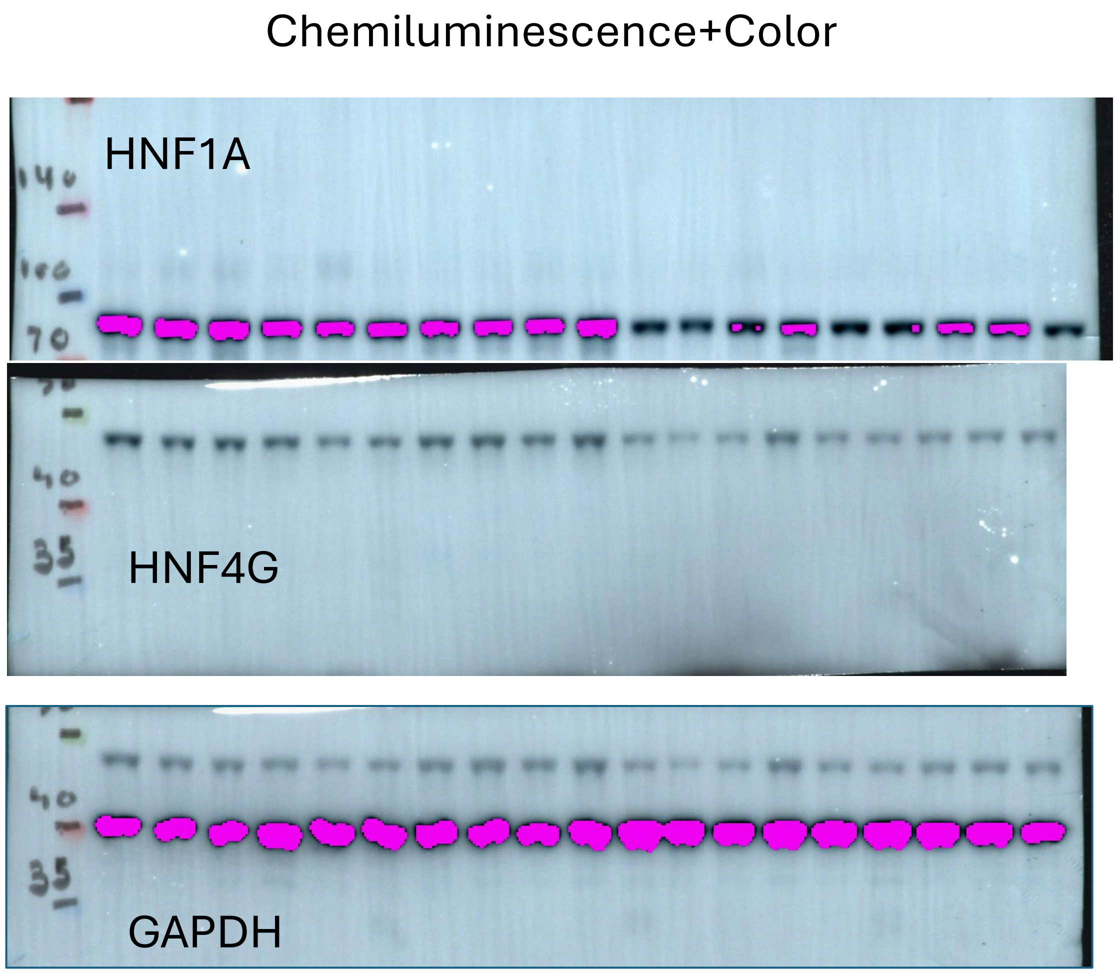
Chemiluminescence+Color
140
100
70
HNF1A
40
35
HNF4G
40
35
GAPDH

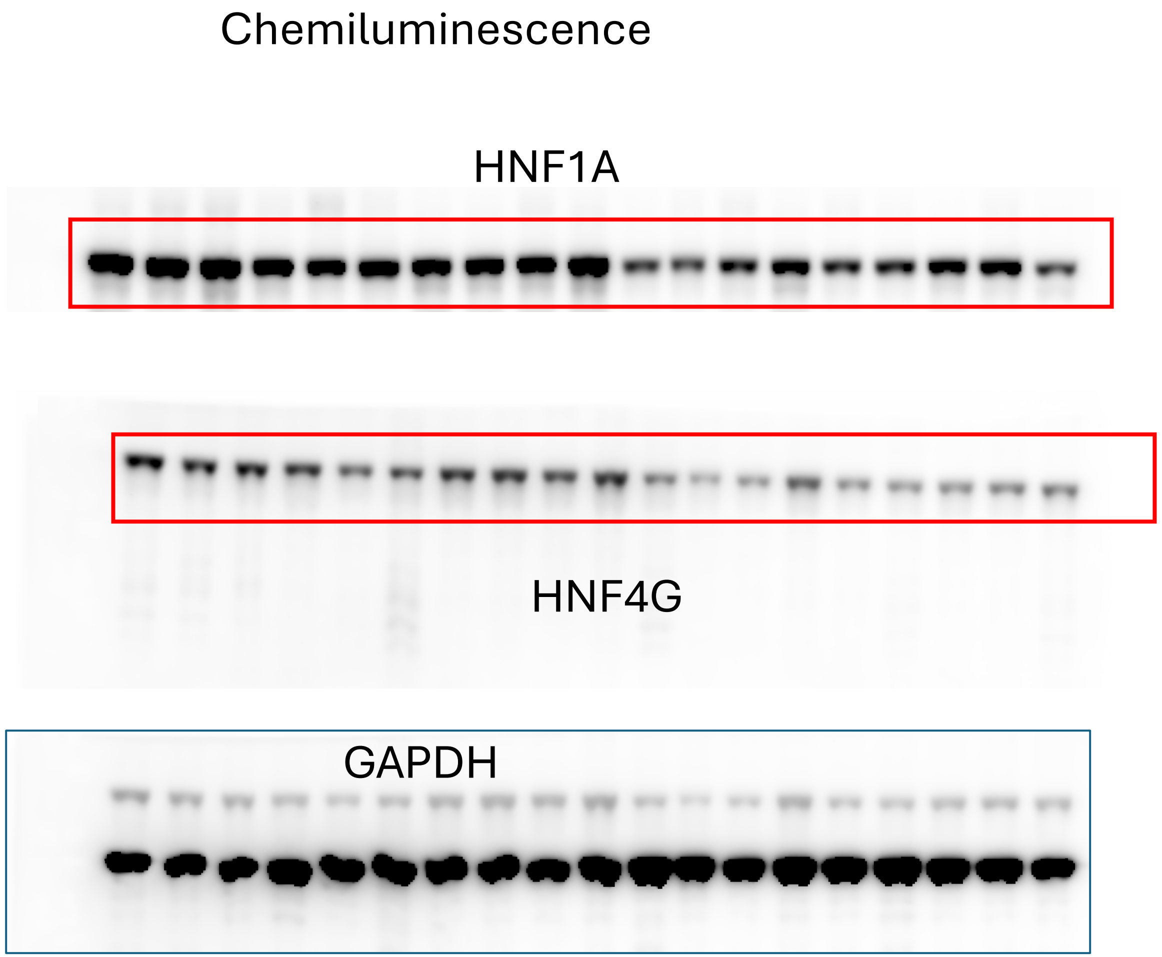
Chemiluminescence
HNF1A
HNF4G
GAPDH

Figure S5B

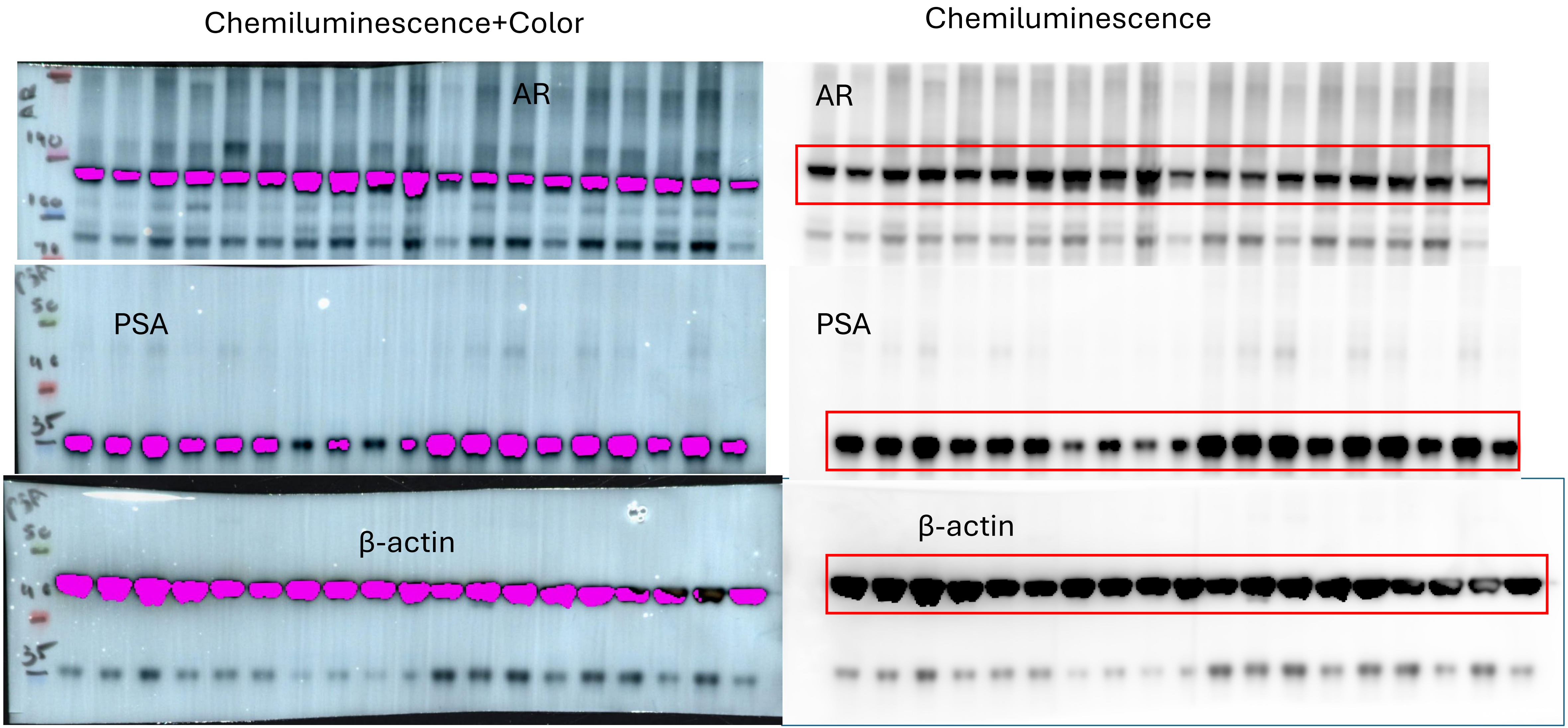
Chemiluminescence+Color
Chemiluminescence
AR
AR
PSA
PSA
β-actin
β-actin

Figure S5B

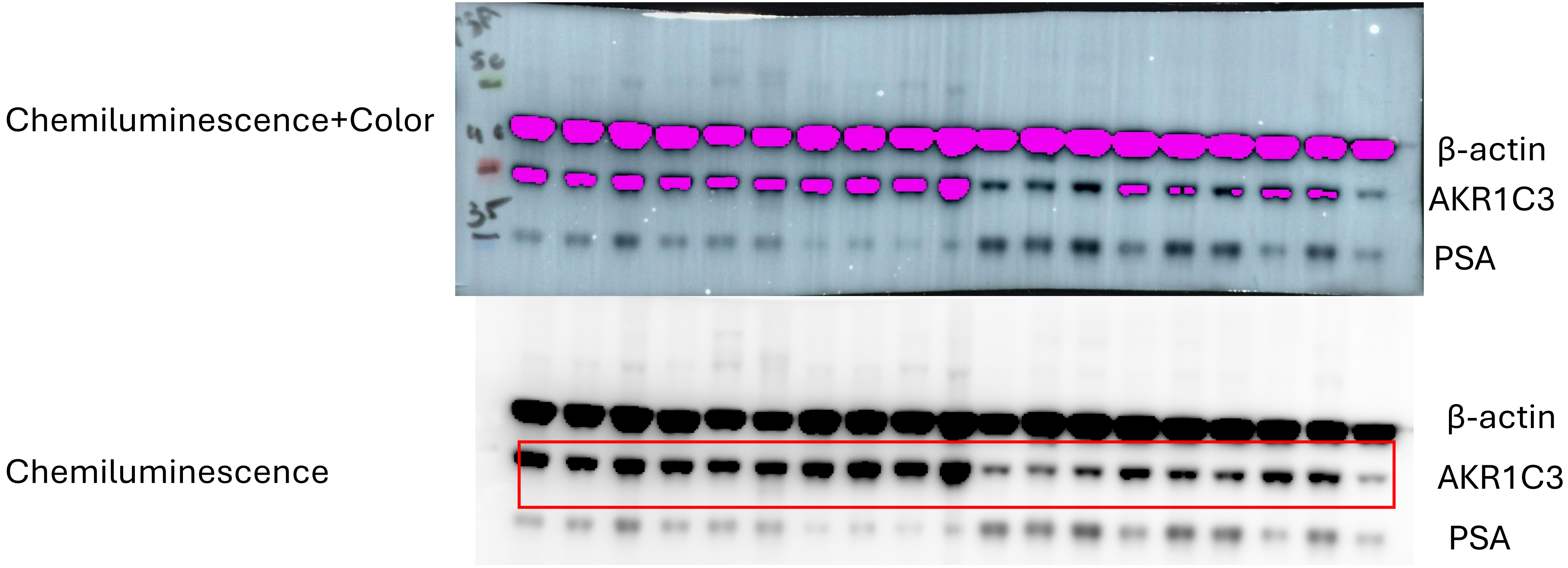
Chemiluminescence+Color
β-actin
AKR1C3
PSA
Chemiluminescence
β-actin
AKR1C3
PSA

Figure 7B

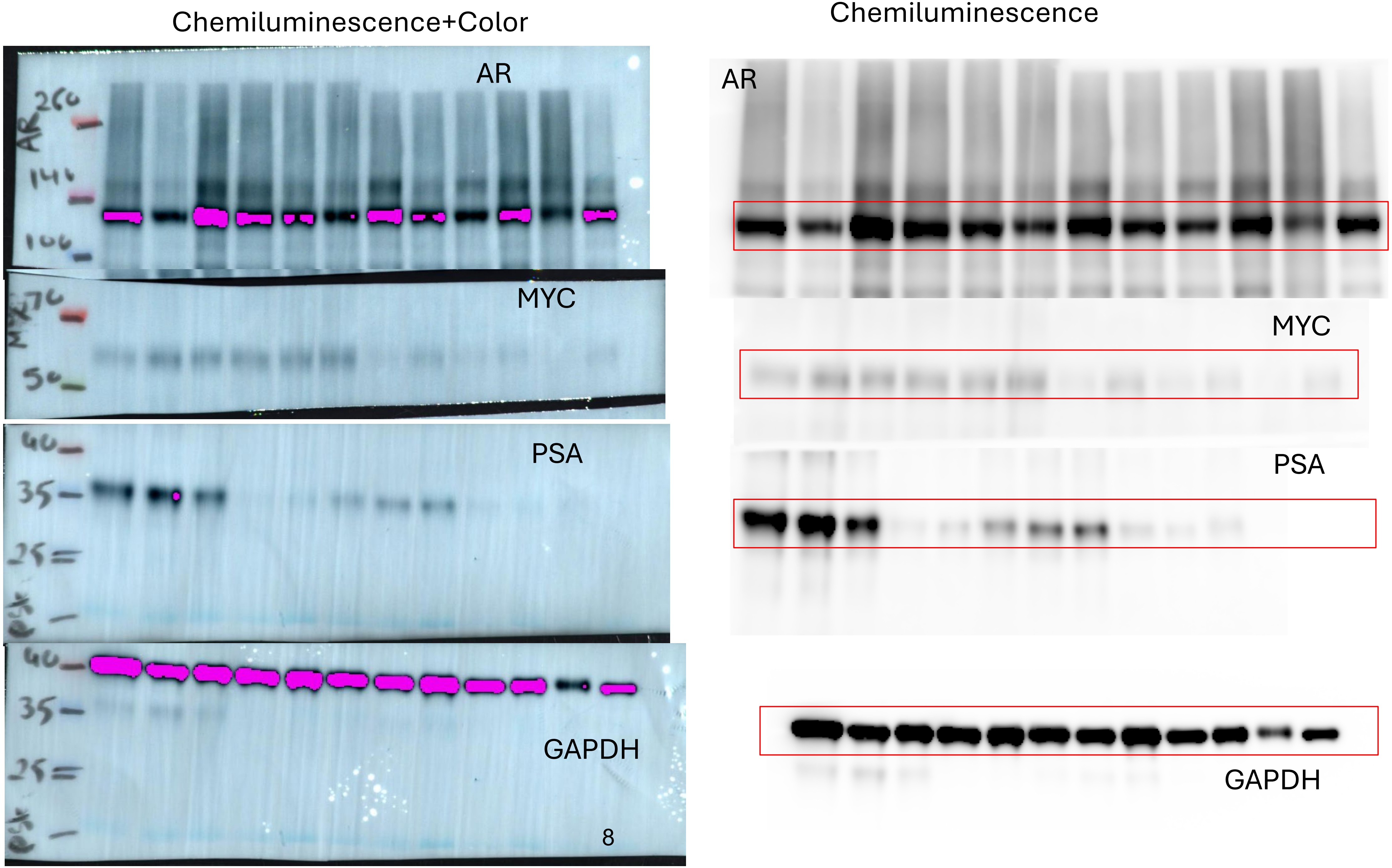

Chemiluminescence+Color
Chemiluminescence
AR
MYC
PSA
GAPDH
AR
MYC
PSA
GAPDH

Figure 7B

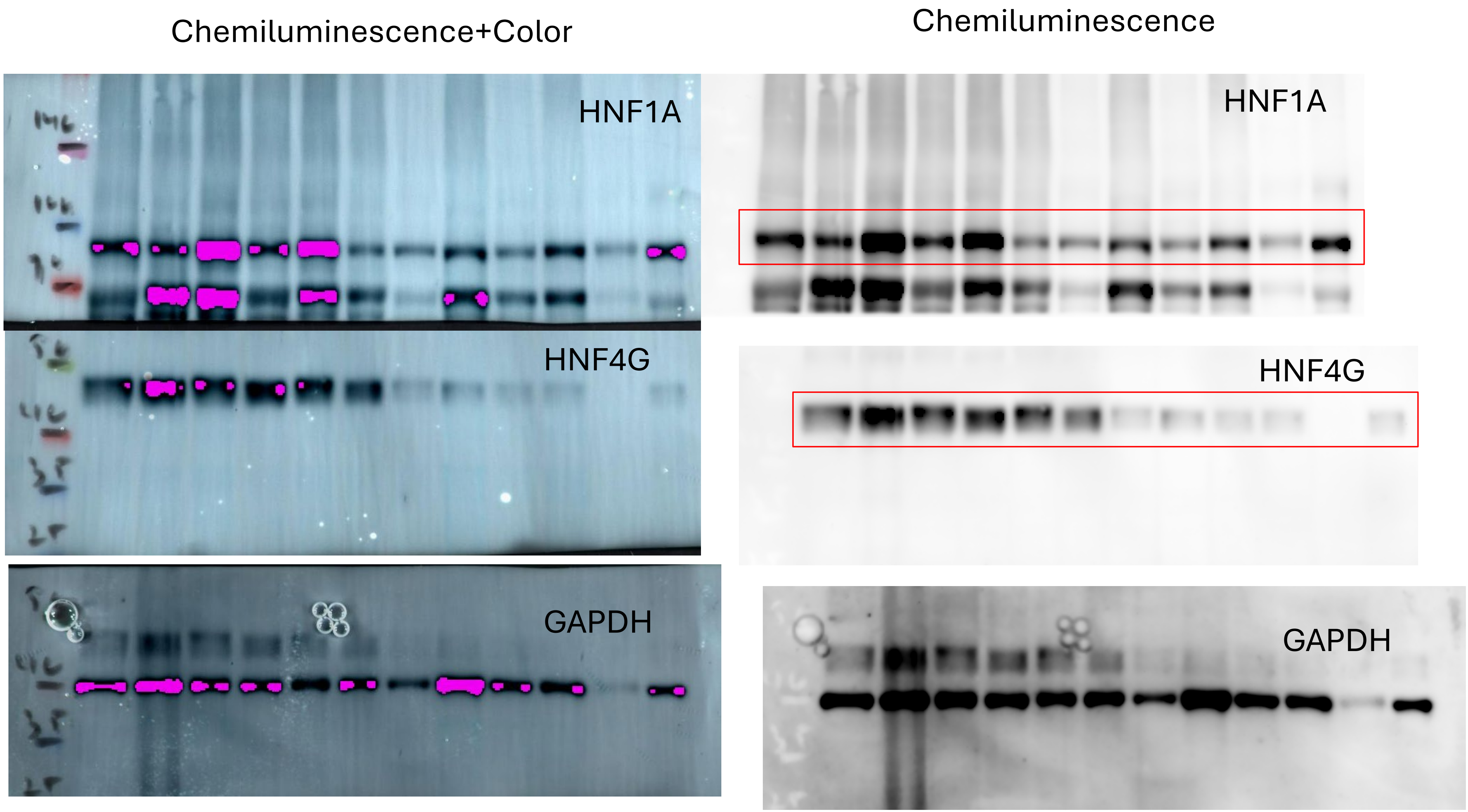
Chemiluminescence+Color
Chemiluminescence
HNF1A
HNF4G
GAPDH
HNF1A
HNF4G
GAPDH

Figure 7E

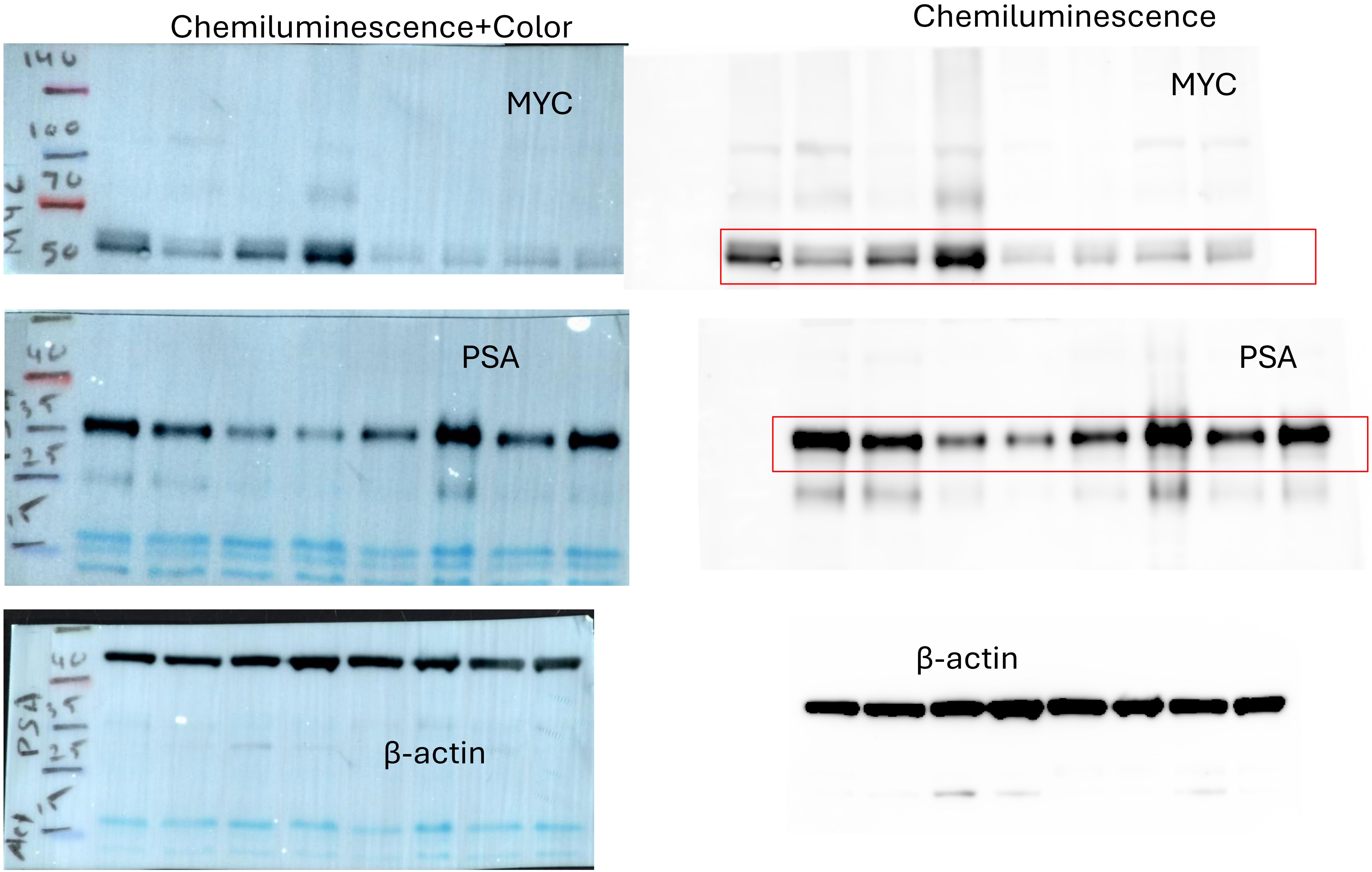
Chemiluminescence+Color
Chemiluminescence
MYC
MYC
PSA
PSA
β-actin
β-actin

Figure 7E

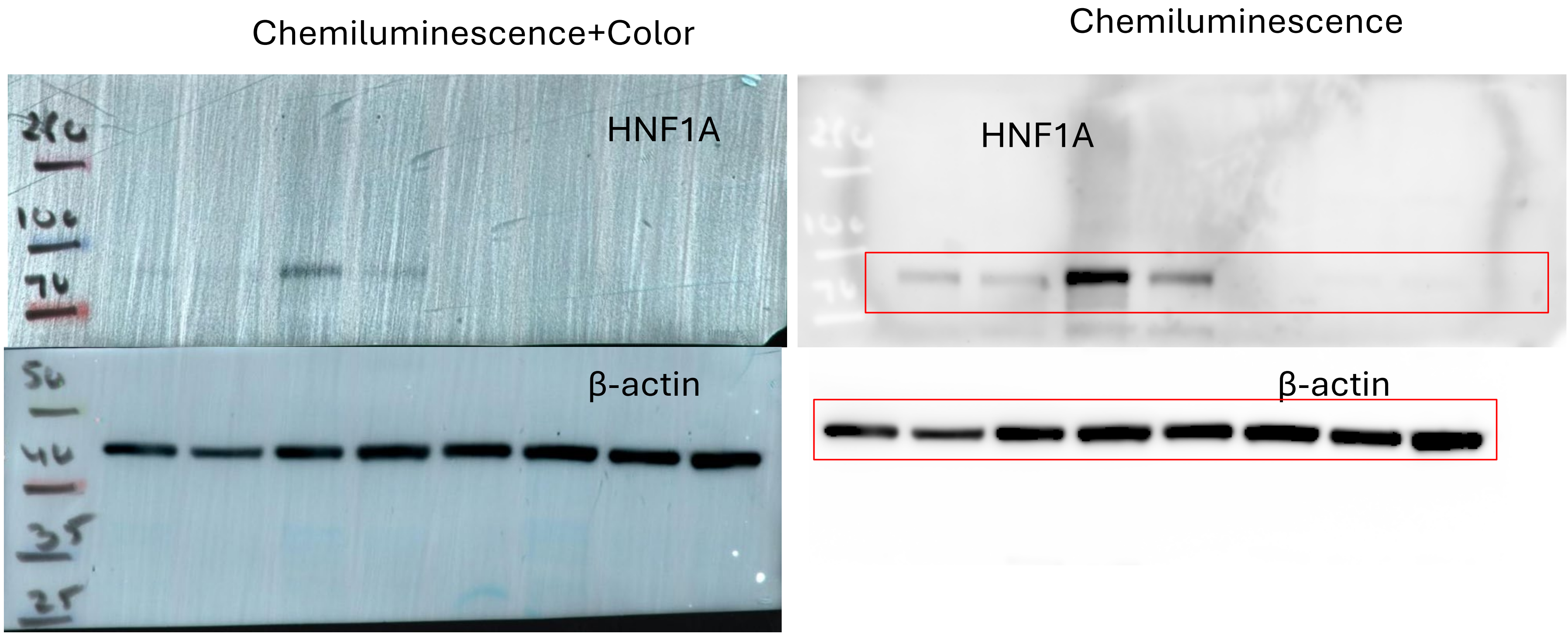

Chemiluminescence+Color
HNF1A
β-actin
Chemiluminescence
HNF1A
β-actin

Figure 7E

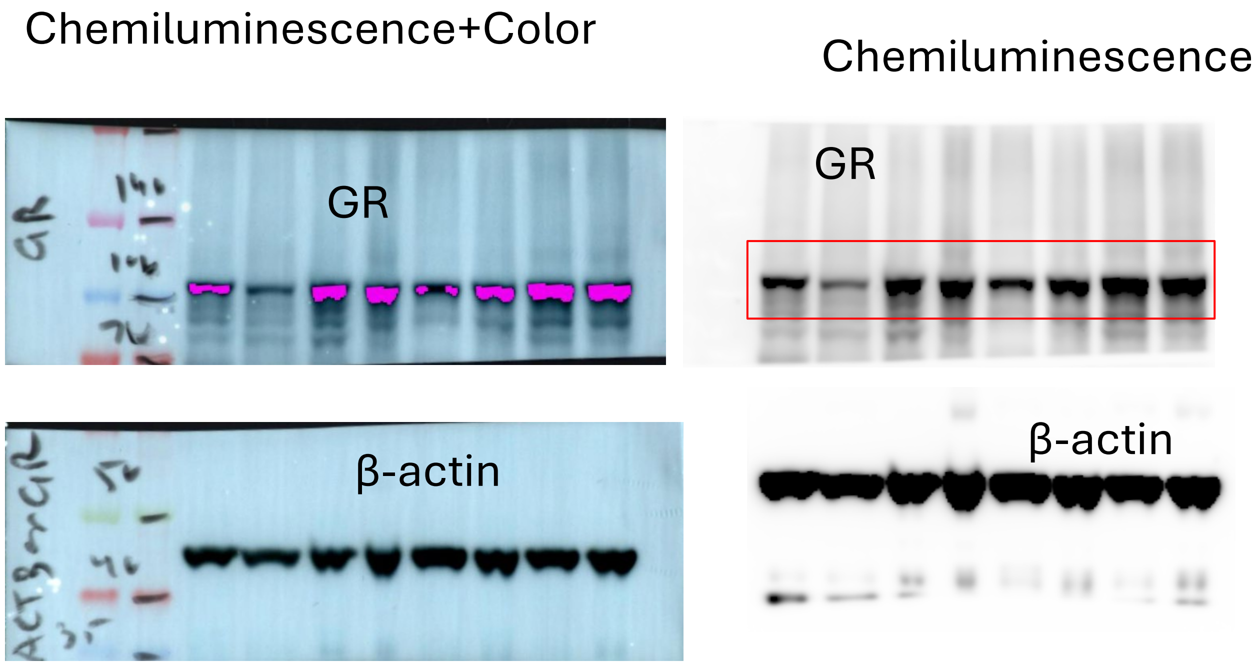
Chemiluminescence+Color
Chemiluminescence
GR
GR
β-actin
β-actin

Figure 7E

Chemiluminescence+Color

Chemiluminescence

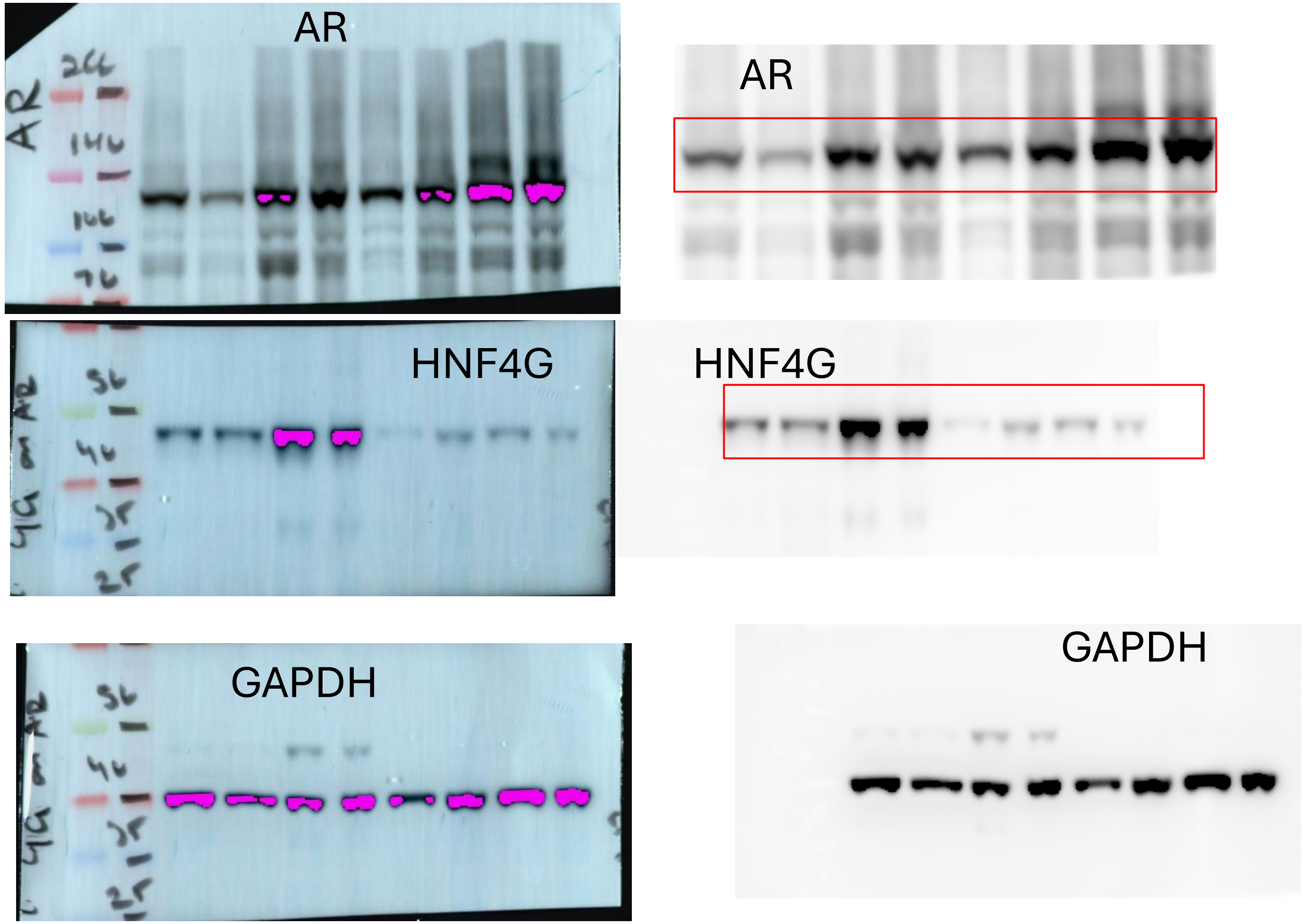

Figure 7H

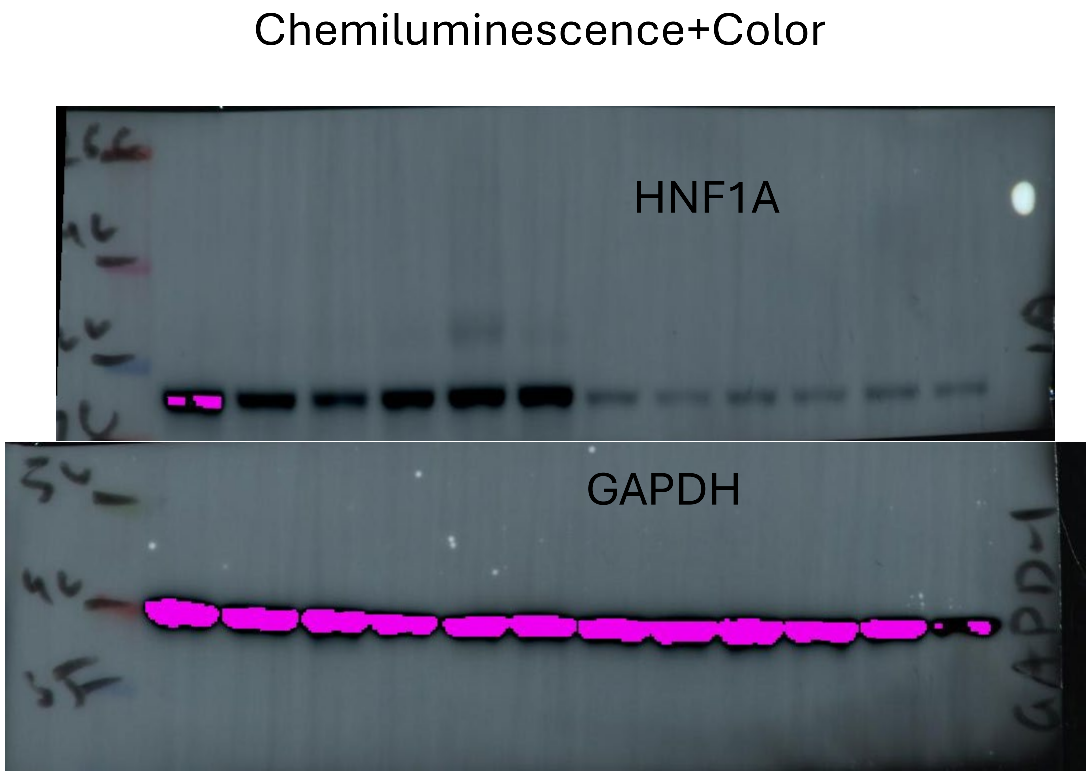
Chemiluminescence+Color
HNF1A
GAPDH

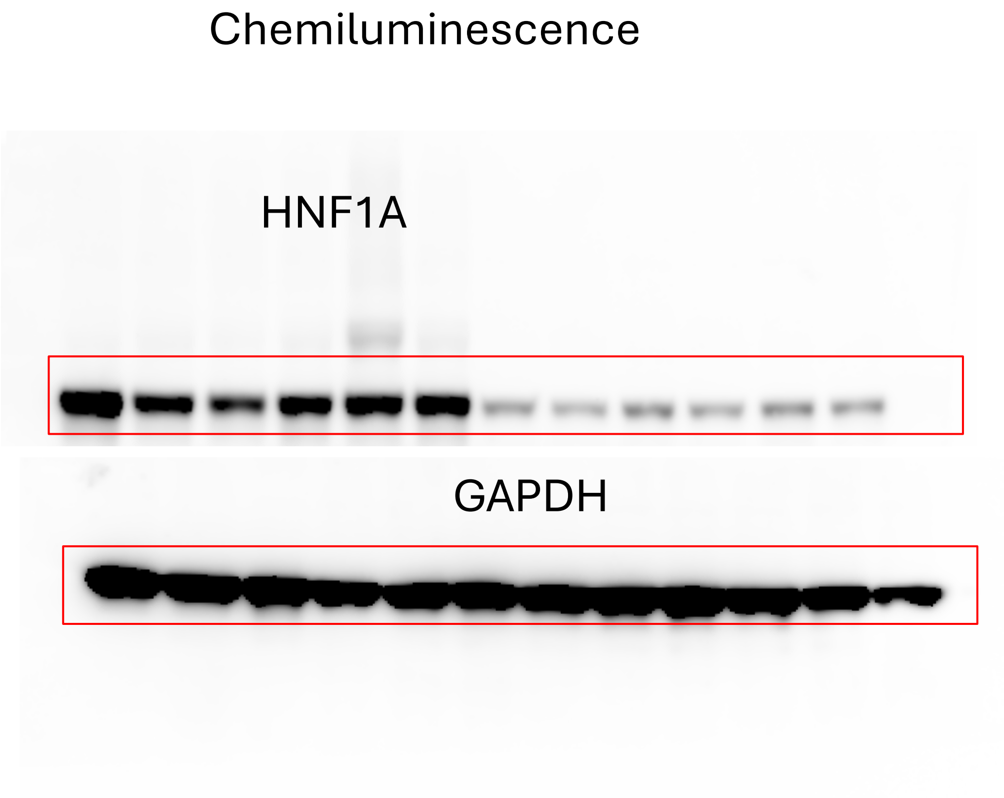
Chemiluminescence
HNF1A
GAPDH

Figure 7H

Chemiluminescence+Color

Chemiluminescence

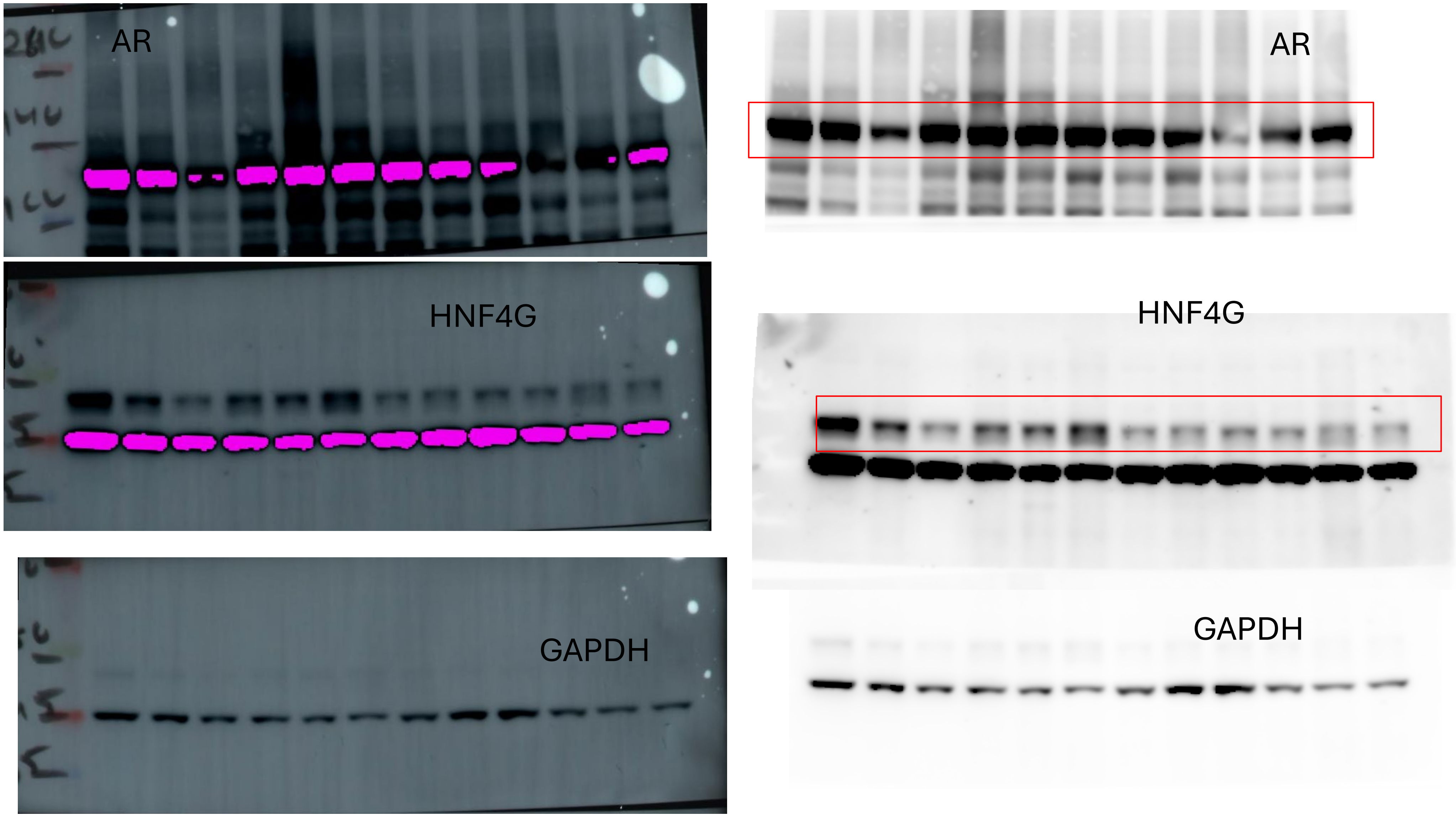

Figure 7H

Chemiluminescence+Color

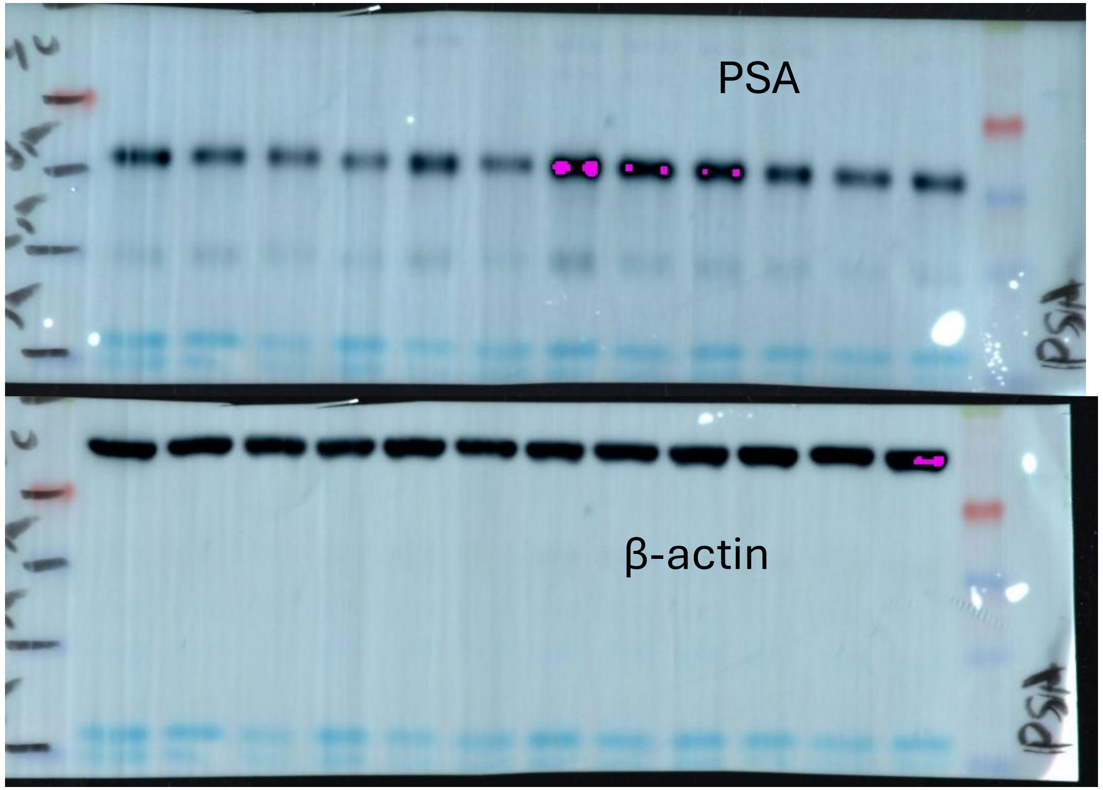

Chemiluminescence

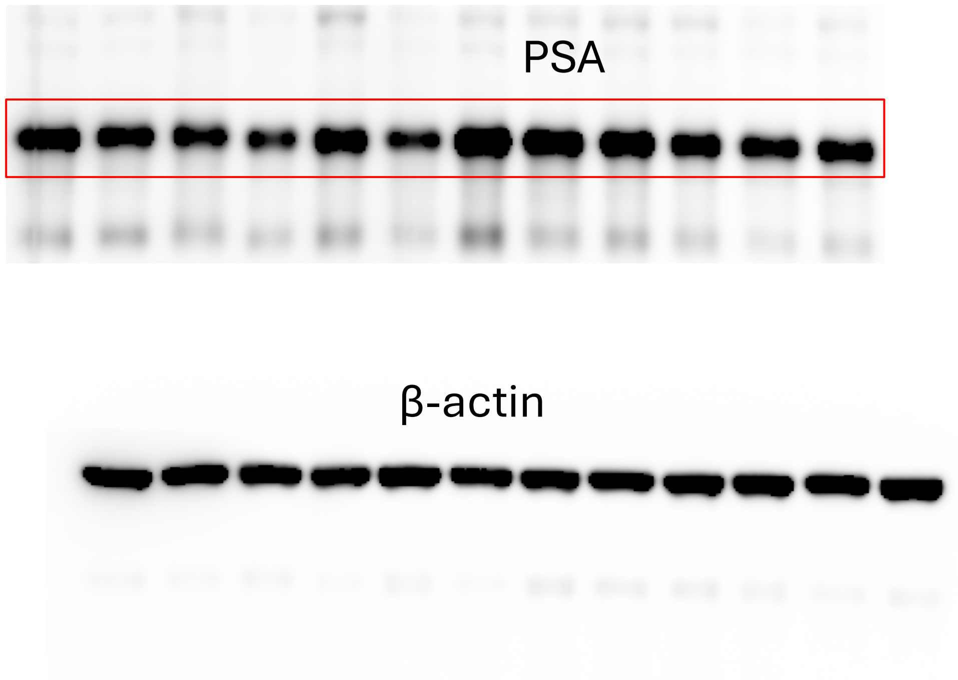

Supplement: Unedited blot and gel images [file jci-135-180378-s030.pdf]
